# A Scalable Strand-Specific Protocol Enabling Full-Length Total RNA Sequencing From Single Cells

**DOI:** 10.3389/fgene.2021.665888

**Published:** 2021-06-03

**Authors:** Simon Haile, Richard D. Corbett, Veronique G. LeBlanc, Lisa Wei, Stephen Pleasance, Steve Bilobram, Ka Ming Nip, Kirstin Brown, Eva Trinh, Jillian Smith, Diane L. Trinh, Miruna Bala, Eric Chuah, Robin J. N. Coope, Richard A. Moore, Andrew J. Mungall, Karen L. Mungall, Yongjun Zhao, Martin Hirst, Samuel Aparicio, Inanc Birol, Steven J. M. Jones, Marco A. Marra

**Affiliations:** ^1^Canada’s Michael Smith Genome Sciences Centre, BC Cancer, Vancouver, BC, Canada; ^2^Department of Molecular Oncology, BC Cancer, Vancouver, BC, Canada; ^3^Department of Medical Genetics, University of British Columbia, Vancouver, BC, Canada

**Keywords:** full-length, total RNA, single-cell, RNAseq, cellenONE

## Abstract

RNA sequencing (RNAseq) has been widely used to generate bulk gene expression measurements collected from pools of cells. Only relatively recently have single-cell RNAseq (scRNAseq) methods provided opportunities for gene expression analyses at the single-cell level, allowing researchers to study heterogeneous mixtures of cells at unprecedented resolution. Tumors tend to be composed of heterogeneous cellular mixtures and are frequently the subjects of such analyses. Extensive method developments have led to several protocols for scRNAseq but, owing to the small amounts of RNA in single cells, technical constraints have required compromises. For example, the majority of scRNAseq methods are limited to sequencing only the 3′ or 5′ termini of transcripts. Other protocols that facilitate full-length transcript profiling tend to capture only polyadenylated mRNAs and are generally limited to processing only 96 cells at a time. Here, we address these limitations and present a novel protocol that allows for the high-throughput sequencing of full-length, total RNA at single-cell resolution. We demonstrate that our method produced strand-specific sequencing data for both polyadenylated and non-polyadenylated transcripts, enabled the profiling of transcript regions beyond only transcript termini, and yielded data rich enough to allow identification of cell types from heterogeneous biological samples.

## Introduction

Bulk RNA sequencing (RNAseq) is commonly used to study the average gene expression of cells within a population. The relatively recent introduction of single-cell RNAseq (scRNAseq) has provided insights into cell-level heterogeneity in biological samples in developing tissues (e.g., Scialdone et al., 2016) and tumors (e.g., Tirosh et al., 2016) at unprecedented resolution. It has become clear that to accurately assess the spatial and temporal patterns of gene expression in healthy and diseased cells, the profiling of samples at a single-cell resolution is vital.

The first step of scRNAseq is the isolation of individual cells, where capture efficiency remains a significant challenge. Several existing approaches include flow cytometry, limiting dilution, laser capture microdissection, and microfluidic techniques (Kolodziejczyk et al., 2015; Ziegenhain et al., 2017). Others involve the trapping of single cells within droplets followed by on–bead or in-droplet molecular barcoding of cells (Kolodziejczyk et al., 2015; Ziegenhain et al., 2017). Approaches differ in their cost, efficiency, starting material type and number of cells required while low capture efficiencies and cell size restrictions of microfluidic approaches remain a challenge (Kolodziejczyk et al., 2015; Ziegenhain et al., 2017; Cao et al., 2017, 2019). To address some of these shortcomings, Cao et al. (2017) developed a combinatorial cell indexing approach uniquely free of both single cell isolation or compartmentalization techniques (Cao et al., 2017, 2019).

Regardless of the method used for single cell isolation, scRNAseq protocols are further limited by the amount of RNA in single cells. Since the first scRNAseq method was published by Tang et al. (2009), several approaches have been developed to improve RNA capture efficiency. Even so, scRNAseq protocols are generally limited to capturing only the 3′or 5′ ends of transcripts (Kolodziejczyk et al., 2015) and therefore remain best suited for transcript counting, but not for examining transcript structures such as splice variants and fusion transcripts, as are often found in cancers. Furthermore, protocols tend to capture only polyadenylated (polyA^+^) transcripts and therefore exclude non-polyadenylated (polyA^–^) transcripts, including some non-coding RNAs. Finally, commonly used scRNAseq protocols do not provide strand-orientation information. Discriminating sense and antisense overlapping transcripts has been important in studies of antisense expression (e.g., Balbin et al., 2015).

The SMART-seq protocol, which employs the Fluidigm C1 System (Durruthy-Durruthy and Ray, 2018), yields data appropriate for full-length transcript analyses but only for polyA^+^ mRNAs. Recently, Hayashi et al. (2018) reported a scRNAseq protocol that also employed the Fluidigm C1 System, but as it only allowed for processing of up to 96 cells per run, sensitivity to minor cell populations is low (Hayashi et al., 2018), thus constraining the technique to samples with limited heterogeneity. Moreover, both protocols are strand-agnostic, which is known to lead to inaccurate transcript quantification and does not readily allow for studies of anti-sense RNA biology (Mills et al., 2013; Sigurgeirsson et al., 2014; Zhao et al., 2015).

To better profile gene expression at single-cell resolution, a high-throughput, strand-specific protocol with minimal 3′ or 5′ bias that extends sequence results beyond polyA^+^ RNA is needed. Here, we report a method that addresses the aforementioned limitations, and demonstrate its capacity to process over 1,000 cells per run. This protocol enables full-length, strand-specific sequencing of total RNA at single-cell resolution, providing researchers with an avenue for a more complete analysis of gene expression in heterogeneous biological samples.

## Materials and Methods

### Cell Line and RNA Samples

**FIGURE 1:**
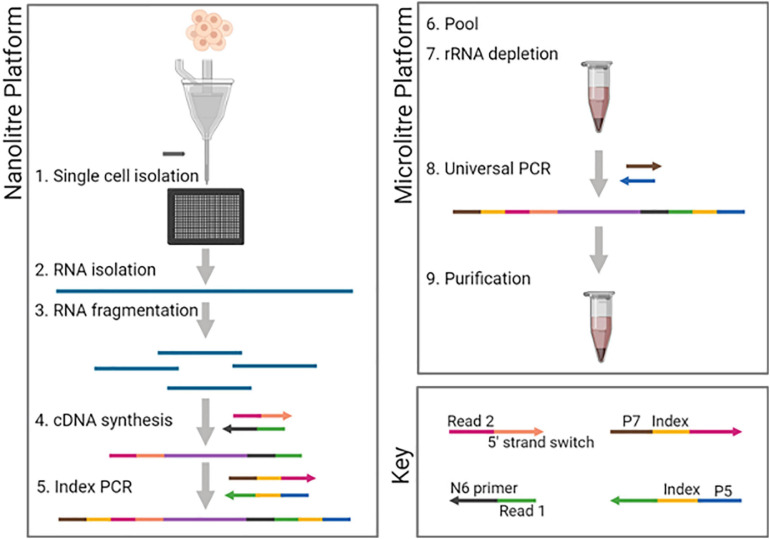
DLP-scRNAseq workflow. Following single-cell isolation using the CellenONE automated cell spotter and lysis, RNA was fragmented using magnesium ion-dependent heating. Adapters containing 5′- and 3′-end sequencing primer targets were introduced sequentially as part of the cDNA synthesis steps, thereby achieving strand-specificity. Cell-specific barcodes were introduced in the first round of PCR (Index PCR). All steps up to Index PCR were performed in nanoliter-scale wells (Nanoliter platform). PCR products were then pooled and subsequent steps including rRNA depletion were performed in 96-well plate format (Microliter platform). Figure was created using biorender.com.

Universal Human Reference (UHR) total RNA was obtained from Stratagene (Cat. No.740000) and quantified using the Agilent RNA 6000 Nano Kit (Cat. No.5067-1511). For the input titration experiments shown in Figure 1, UHR was spiked with External RNA Controls Consortium (ERCC) spike-in mix 1 from Ambion (Cat. No.4456740) where 0.02 μL of the spike-in mix (∼1.035 moles) was used per 1 μg UHR total RNA. For the single-cell experiments, an equivalent of 1 μL of one million-fold dilution of the ERCC mix 1 stock (∼0.1 attomoles) was used per well. The immortalized Normal Human Astrocyte (NHA) cell line (Sonoda et al., 2001) was obtained from Applied Biological Materials (ABM) Inc (T3022; Richmond, BC, Canada) while the Human Peripheral Blood Mononuclear Cells (PBMCs) were purchased from STEMCELL Technologies (Cat. No.70025.1).

### Sample Preparation for RNAseq

#### Standard and Modified RNaseH rRNA Depletion

Standard RNaseH rRNA depletion was applied to 1–10 ng of total RNA as described previously (Haile et al., 2017a, 2019) except that half of the rRNA probe amount was used. Upstream DNase I treatment was omitted as the probe removal DNase treatment step that is integrated into the rRNA depletion kit was found to be sufficient for removing residual gDNA contamination.

For modified RNase H-based rRNA depletion, unpurified RNA following rRNA depletion was incubated at 95°C for 10 min without EDTA to heat-inactivate the DNase and to fragment the RNA in a Mg^2+^-dependent manner (Mg^2+^ is part of DNase reaction buffer as a cofactor for the enzyme). The amount of rRNA probe used was half of the standard amount for 1–10 ng total RNA and fivefold less than the standard amount for < 1 ng total RNA. Following the rRNA depletion, DNase I treatment, and RNA fragmentation steps, first-strand cDNA synthesis was performed directly without purification to remove contaminants from upstream reactions. The first-strand buffer amount was adjusted to account for the buffers in the upstream reactions and was spiked with DTT to a final concentration of 2.5 mM as is standard for first strand cDNA synthesis. cDNA synthesis and library construction steps were performed as described previously (Haile et al., 2019). PCR was performed using 15 and 18 cycles for 1–10 ng and 0.1–0.25 ng total RNA input, respectively.

#### SMART-Seq_v4

SMART-Seq v4 Ultra Low input RNA for sequencing (Cat. No. 634888; Takara Bio Inc) was used according to the manufacturer’s recommendations. PCR was done using 14 cycles for 0–250 pg total RNA input, 10 cycles for 1 ng, and 7 cycles for 5–10 ng. Following purification of the PCR reactions, 150 pg of amplified cDNA was used for library construction using the Nextera XT DNA Library Preparation Kit (Cat. No. FC-131-1024; Illumina) as per the manufacturer’s recommendations.

#### PolyA-Based RNAseq and Exome RNAseq

PolyA-based libraries were constructed as described previously (17). For exome RNAseq, total RNA was directly used for cDNA synthesis and library construction steps as described previously (Haile et al., 2017b). PCR was done using 15 cycles to amplify 0–250 pg RNA and 13 cycles to amplify 1–10 ng. Following purification of the PCR reactions, 500 ng of amplified libraries were used for exome capture as described previously (Cieslik et al., 2015).

#### SMARTer Bulk Total RNAseq

SMARTer^®^ Stranded Total RNAseq Kit v2–Pico Input Mammalian (Cat. No. 634413; Takara Bio Inc.) was used as per the manufacturer’s instructions when the starting material was total RNA. PCR was done using 16 cycles of PCR to amplify 0–250 pg of RNA, 14 cycles to amplify 1 ng, and 12 cycles to amplify 5–10 ng.

For bulk RNAseq, 10–500 cells, based on hemocytometer cell counting, were first washed and resuspended in 2 μL of 1× PBS and were mixed with 6 μL of 1× lysis buffer (Cat. No. 635013; Takara Bio Inc) containing 0.5% RNase inhibitor (Cat. No. 635013; Takara Bio Inc). The rest of the steps were performed according to the manufacturer’s instructions in the supplementary Pico v2 protocol for intact-cell inputs (Takara Bio Inc). Prior to rRNA depletion, 5 cycles of PCR were used to amplify cDNA fragments. Following rRNA depletion, 18 cycles of PCR were used for 10 cells and 14 cycles for 500 cells.

#### Direct Library Preparation (DLP)-Based Single Cell Total RNAseq (DLP-scRNAseq)

Cell spotting was performed using the cellenONE (Cellenion) platform as previously described for the Direct Library Preparation Plus (DLP+) single-cell genome sequencing protocol (Laks et al., 2019). For single cells, the upstream RNAseq preparation steps including cell lysis, RNA fragmentation, cDNA synthesis and adapter addition were performed as described above for the SMARTer bulk protocol, but generally with volumes in nanoliters as opposed to microliters. The step-by-step details of the protocol are attached in Supplementary Text File (pages 5–29). For optimal spotting of reaction mixes other than the lysis/fragmentation mix, 0.05% Tween-20 was spiked into the reactions. Reaction mixes and primers were filtered using spin-x columns (Cat. No. CLS8162; Merck) whenever spotting proved to be problematic. All steps up to and including the introduction of cell-specific indices during the first round of PCR (pre-rRNA depletion), were performed in nanoliter volumes using Takara Smart Chips (Takara Bio Inc). These arrays consist of a 72 × 72 (5184) well layout each of which able to hold a volume of approximately 100 nl. After 5 cycles of the first round of PCR, the chip was inverted and spun down to pool all reactions into one tube. Subsequent steps were performed according to the SMARTer Stranded Total RNAseq Kit v2–Pico Input Mammalian manufacturer’s instructions.

The SMARTer^®^ kit comes with indexing primers that allow the barcoding of a maximum of 96 samples. To increase the number of cells that could be processed, we designed our own barcodes based on the following requirements: (1) the random primer and strand-switching oligos were to be anchored to Illumina sequencing primer sequences, and (2) primers used for the first round of PCR must have complementary sequences to the Illumina sequencing primer anchors internally, followed by indices in the middle, and P5/P7 priming sites at their distal ends. We thus designed 72 × 72 dual indexing primers enabling 5184 unique cell-specific barcodes (Supplementary Table 1).

### Sequencing and Bioinformatic Analysis

We generated the following libraries using the DLP-scRNAseq: 402 single NHA cells with the same indexing primers, 92 single NHA cells with unique cell-specific primers, and triplicates of no-cell negative controls and positive control 5 pg UHR total RNA. These libraries were pooled into one tube, which we referred to as the nano-pool. We also prepared the following libraries in the microliter platform: 10 NHA cells in bulk, 500 NHA cells in bulk as well as a single replicate of 4 ng total UHR.

The DLP-scRNAseq NHA pool (0.5×), the bulk SMARTer NHA 500 cells (0.2×), the bulk NHA 10 cells (0.2×), and the bulk UHR (0.1×) were pooled and sequenced on one lane of an Illumina HiSeq 2500 flowcell (paired end 75 bp).

#### JAGuaR Alignment

Sequence analysis was performed as described previously (Haile et al., 2017a, b, 2019), and briefly involved alignment of reads to the hg19 reference genome in combination with Ensembl 69 gene models using the JAGuaR junction-aware alignment pipeline (Butterfield et al., 2014) using the “mem” alignment option in place of “aln.” Gene expression values were calculated exactly as described in Haile et al. (2017a, b, 2019). Briefly, the read chastity status was first marked with custom scripts and duplicates were marked with Sambamba 0.5.5 (Tarasov et al., 2015). Reads were then split into positive- and negative-strand BAM files, analyzed for depth of reads after converting to wig files, and finally reads per kilobases per million (RPKM) values were generated from these counts.

When comparing results for non-single-cell libraries, we control for depth-related variables by down-sampling the original BAM files to obtain approximately equal numbers of reads for each library. Down-sampled read alignments were subsequently enumerated to generate an expression matrix of sample-by-gene RPKM estimates that were then used in correlation analyses to evaluate the similarities in expression profiles across samples and protocols.

Sequencing data is deposited at Sequence Read Archive (SRP286135).

#### STAR Alignments

RNA read alignments were performed with STAR 2.7.3a (Dobin et al., 2013) in 2-pass mode after detecting adapter sequence using bbmerge (Bushnell et al., 2017) and trimming with cutadapt version 1.16 (Chen et al., 2018). TPM expression estimates were generated from the STAR alignments using Stringtie (Pertea et al., 2015).

#### Comparison With qPCR Data

UHR qPCR data from the MicroArray Quality Control project (GSE5350) (MAQC Consortium et al., 2006) were downloaded for comparison to our expression results. Using samples GSM129638-GSM129641, expression estimates were matched by gene name between our RPKM values and the published qPCR estimates. Each sample was correlated with all four replicate qPCR data sets, from which a median Pearson correlation was calculated.

#### Exon-Level Analysis

For exon analysis, BAM files were generated from aligning reads (read 1&2 lengths of 69 bp for single-cell libraries and read 1&2 length of 75 bp for bulk libraries) using JAGuaR as described above.

Exon quantification was performed for full exons as well as partial exons that fell within the 3′ and 5′ untranslated regions (UTRs) of annotated transcripts. Partial exon means only part of an exon falls within either the 3′ or 5′ UTR of a transcript. The analysis was performed using the following R packages: *GenomicFeatures* (v1.26.4), *GenomicRanges* (v1.26.4), *Rsamtools* (v1.26.2), *IRanges* (2.8.2), and *GenomicAlignments* (v1.10.1). All exon start and end locations and their associated transcript and gene IDs were retrieved from the Ensembl databases using the functions *makeTxDbFromUCSC* and *exons* from *GenomicFeatures*. The functions *fiveUTRsByTranscript* and *threeUTRsByTranscript* from *GenomicFeatures* were used to extract the start and end coordinates of full or partial exons that constituted the 3′UTR or 5′UTR regions of each transcript.

Non-duplicate paired-end reads were imported from the BAM files using *readGAlignmentPairs* from *GenomicAlignments*. A second filtering step was applied to keep only reads that aligned to genomic locations that did not fall exclusively within 100 bp of the start and end of each chromosome. The second filtering step was applied to avoid the confounding effects of telomeric repeats on read mapping. The number of reads that overlapped with each exon, or each exonic region within the 3′UTR or 5′UTR of transcripts, was quantified using the *countOverlaps* function from the *IRanges* package. A read could map to multiple exons or exonic regions if its genomic coordinates overlapped with the coordinates of more than one region. For expression-based comparisons of expression levels, exon counts were normalized for sequencing depth using *calcNormFactors* and converted to RPKM using the *rpkm* function from *edgeR* v3.24.3.

#### PBMC Clustering Analysis

For the PBMC clustering analysis, the fastq file for the 10X PBMC data was obtained from the 10X website^1^, and the CellRanger pipeline (v3.0.2) was used to obtain a count matrix for 1,223 cells, aligning to hg19 (v3.0.0^2^). Data preprocessing was performed in R, based on the count matrices output by HTSeq (Anders et al., 2015) using the JAGuaR-based read alignments (DLP-scRNAseq data) or by the CellRanger pipeline (10X data). For the DLP-scRNAseq dataset, counts from all wells identified as containing a cell (*n* = 517) were combined into a single count matrix. Outliers were identified based on total read counts, total number of genes detected, and the percent of counts coming from ERCC spike-ins for DLP-scRNAseq, as previously described (Lun A.T et al., 2016). For each of these metrics, cells with lower (read counts and genes detected) or higher (percent of counts from ERCCs) than three median absolute deviations from the median were considered outliers (*n* = 56 for the DLP-scRNAseq dataset; *n* = 94 for the 10X dataset). After cell filtering, genes with at least one read count in at least two cells were retained, resulting in a final datasets with 461 cells and 16,642 genes (DLP-scRNAseq) and 1,129 cells and 15,982 genes (10X). Normalization was then applied to all cells using the *scran* package (V1.10.1) (Lun A.T et al., 2016). The *quickCluster* function was used to cluster cells for normalization with min.mean = 0.1 for the DLP-scRNAseq dataset, as suggested for read count data, and 0.01 for the 10X dataset, as suggested for UMI data. Resulting clusters were used as input to the *computeSpikeFactors* (DLP-scRNAseq, with ERCC reads labeled as spike-ins) or *computeSumFactors* (10X) function. These factors were then used in the *normalize* function of the *scater* R package (v1.10.0) to obtain normalized expression values that were used for downstream analyses.

Cell clustering was performed largely as described previously (Lun A.T.L. et al., 2016). Highly variable genes (HVGs) were first identified using the *trendVar* function of the *scran* R package with *parametric* set to TRUE, a span of 0.3 for the LOESS fitting, and min.mean set to 0.1 (DLP-scRNAseq) or 0.01 (10X). The *decomposeVar* function was then used to decompose gene-specific variances into biological and technical components, and genes with a biological component >0.1 and a Benjamini-Hochberg-corrected *p* < 0.05 were considered HVGs. Principal component analysis (PCA) was performed using the *parallelPCA* function on the normalized expression matrix containing only HVGs, and 1,000 permutation iterations were performed to identify significant principal components (PCs). Briefly, this function permutes the expression vector for each gene and repeats the PCA to calculate the fraction of variance explained by each PC (up to 100) under a random null model; all PCs from the first PC where the permuted fractions exceed the observed fraction of variance in more than 10% of iterations (the default threshold) are then discarded, and earlier PCs are retained as “significant PCs” (with a minimum of five). A shared nearest neighbor graph (*k* = 15) was then obtained using the *buildSNNGraph* function based on the PCA reduction (with six and seven significant PCs for the DLP-scRNAseq and 10X datasets, respectively), and the *cluster_walktrap* function from the *igraph* R package (v1.2.2) was used to identify clusters.

Marker genes with high expression in individual clusters were identified using the *overlapExprs* function from *scran*, which performs Wilcoxon rank-sum tests between each pair of clusters and then calculates a combined *p*-value using Sime’s method. The tSNE plots used for visualization were obtained using the *Rtsne.multicore* R package (v0.0.99) with perplexity = 50, theta = 0.0, and a maximum of 2,000 iterations, based on the significant PCs described above. The correlation analysis to reference cell types was performed using the *SingleR* (v0.2.0) (Aran et al., 2019) tool in R with the LM22 matrix (Newman et al., 2015) as a reference. For this analysis, normalized expression values from the DLP-scRNAseq dataset were further normalized for gene length using the approach described in Reid et al. (2018).

#### PBMC Alternative Splicing Analysis

Splicing patterns were first quantified in individual cells using BRIE (Huang and Sanguinetti, 2017) and the lenient annotations provided by the tool’s authors (Gencode v19^3^). Differential splicing was then performed between each pair of cells using default parameters. Events with a Bayes factor ≥10 and a difference in the proportion of spliced isoform (ΔPSI) > 0.2 were considered to be differentially spliced (Bray et al., 2016). For each pair of cell types, the total possible number of events was calculated as follows: # of cells in cell type 1 × # of cells in cell type 2 × # of unique transcripts in the annotation file.

To identify cell type-specific alternative splicing events, we first pooled reads from all cells assigned to the same cell type. BRIE was then used to quantify events in each cell type and perform differential splicing analyses between each pair of cell types using default parameters. Events with a Bayes factor ≥10 were considered to be differentially spliced between cell types (Huang and Sanguinetti, 2017) and events that were specific to one cell type (i.e., had a higher or lower PSI than all other cell types) were identified (Supplementary Table 3). Sashimi plots were created using the script provided with the *briekit* tool^4^ using default parameters.

#### ERCC Spike-in Analysis

ERCC alignment and sensitivity analysis were performed using seqtk (default parameters)^5^ to down-sample the fastq files when matching depths were required. Fastp (Chen et al., 2018) was used to detect and trim adapters, after which alignment and gene expression quantification were performed with Kallisto (Falcao et al., 2018). Sensitivity analysis was performed using logistic regression as outlined in Svensson et al. (2017). A Nextflow script orchestrating these operations across folders of fastqs is available at https://svn.bcgsc.ca/bitbucket/projects/RCORBETT/repos/single_cell_rna/browse where the R scripts used to make related figures can also be found.

#### Enhancer and Circular RNA Analyses

Enhancer RNA analysis was performed as described in Hayashi et al. (2018) using the JAGuaR alignments as the starting point. Circular RNA detection was performed with CIRIquant (Zhang et al., 2020) for which a Nextflow script and associated R notebook are available at https://svn.bcgsc.ca/bitbucket/projects/RCORBETT/repos/single_cell_rna/browse.

## Results and Discussion

Here, we address limitations of current scRNAseq approaches, pursuing two aims: (1) identification and optimization of a strand-specific scRNAseq protocol that offers the potential of full-length transcript analysis of both polyA^+^ and polyA^–^ RNAs on Illumina sequencing instruments, and (2) the potential for automation of such a protocol on a platform that allows for high-throughput processing of various cell types with acceptable recovery of single cells and sequencing data quality.

### Requirements for a Strand-Specific Total RNA scRNAseq Protocol

Random priming of cDNA synthesis was chosen to enable total RNA sequencing, the result of which required both removal of ribosomal RNAs (rRNAs) without loss of cell-specific indexing, and the generation of small sequencing template fragments appropriate for analysis on short-read sequencers. To achieve such fragments, the protocol design incorporated RNA fragmentation. From there, steps leading up to single cell-specific indexing were envisioned as occurring in one reaction vessel, without the need for purifications between protocol steps. The cDNA synthesis step was viewed as the earliest opportunity for cell-specific indexing, and so we preferred the possibility of performing rRNA depletion after cDNA synthesis.

We first conducted a literature search for protocols that met these requirements (Supplementary Figure 1A), and identified or developed three protocols that met these criteria. The first protocol, hereafter referred to as SMARTer, is based on the SMARTer^®^ Stranded Total RNAseq Kit (Takara Bio Inc). In this protocol, rRNA depletion relies on hybridization following the PCR amplification of cDNA fragments. Library construction is not ligation-based, as the introduction of priming sites for Illumina sequencing is integrated into the cDNA synthesis and amplification steps. The second protocol is a variation of an exome RNAseq method that was reported previously for bulk RNAseq (Cieslik et al., 2015). rRNA depletion is done using exome capture and occurs following PCR amplification of adapter-ligated cDNA fragments. The disadvantage of this protocol is that recovered transcripts were limited by probe sets matching annotated exons: transcripts lacking probe sets could not be recovered.

Previously, we showed that the RNaseH rRNA depletion protocol was optimal for low input RNA (Haile et al., 2017a, b, 2019); however, that protocol involved a purification step following rRNA depletion, which occurred prior to cDNA synthesis. We modified this protocol by removing the purification step, thereby providing a third scRNAseq protocol for evaluation (referred to as the Modified RNaseH protocol). We also generated data using the SMART-Seq v4 (SMART_v4) Ultra Low input RNA for sequencing (Takara Bio Inc.), the latest commercial version of the Smart-seq2 protocol that is commonly used for scRNAseq (Picelli et al., 2013). However, this protocol does not meet the requirements mentioned above since it is strand-agnostic, is restricted to poly-A RNAs and is of smaller scale (maximum of 96 cells). We used these data as “gold standard” comparators to the data generated using other protocols, as described below.

We performed comparative analyses of the four protocols described above using Universal Human RNA (UHR) as total RNA input. UHR was spiked with synthetic RNAs from the External RNA Control Consortium (ERCC) at a constant proportion of the input amount to evaluate how well the observed RNA levels correlated with those expected theoretically (External RNA Controls Consortium, 2005). The SMART_v4 protocol and the standard RNaseH rRNA depletion protocol (Haile et al., 2017a, b, 2019) served as our gold standards. Libraries were generated from total RNA input amounts ranging from 100 pg to 10 ng. Except for SMART_V4 and standard RNaseH, where one reaction was used for each of the indicated total RNA input amounts, duplicates were used for all the other protocols for each of the input amounts. Data from various post-sequencing and alignment metrics and expression comparisons are presented in Supplementary Figures 2–9 and are summarized in Supplementary Figure 1B. We used the JAGuaR junction-aware alignment pipeline (Butterfield et al., 2014) for sequence analysis. Compared to STAR, we found that this pipeline enabled a higher mappability of reads to the human reference genome (Supplementary Figure 2A) and a higher sensitivity in the detection of genes (Supplementary Figure 2B) for all the libraries that were generated using the four protocols we described above.

The proportion of reads that aligned to the human genome reference (other than ribosomal RNA and mitochondrial RNA reads) was lowest for the modified RNaseH protocol (as low as 45% vs. >82% for the other protocols) with minimal differences between the other protocols (Supplementary Figure 3). The unaligned reads for the RNaseH protocol appear to result predominantly from microbial contamination. The non-exonic content was lowest for the exome and SMART_V4 protocols (<8 and < 6%, respectively, vs. > 46% for the other protocols) (Supplementary Figure 4). Consistent with a previous report (Ziegenhain et al., 2017), sensitivity of transcript detection and diversity were highest for the SMART_v4 protocol (Supplementary Figures 1B, 5) but these advantages came at the cost of quantitative accuracy of transcript levels as demonstrated by lower expression correlation values with expected levels of ERCC transcripts, UHR expression values obtained using the standard RNaseH and polyA RNAseq protocols, and expression values of 1,000 genes that were previously (MAQC Consortium et al., 2006) quantified using qPCR, especially when compared with the SMARTer protocol. The SMARTer protocol gave the highest base error rate (Supplementary Figure 1B) which appeared to be due to artifacts introduced at strand-switch sites (Supplementary Figure 6). The proportion of properly paired reads for the SMARTer protocol (mean = 78%) was lower than that of the RNaseH protocol (mean = 89%) but higher than that of the SMART_v4 protocol (mean = 70%) (Supplementary Figure 1B). Overall, the SMARTer protocol displayed higher accuracy in representing quantitative expression based on ERCC transcripts (Supplementary Figures 1B, 7; lower panel), comparison with UHR expression values obtained using the standard RNaseH and polyA RNAseq protocols (Supplementary Figures 1B, 7; upper panel), and relative to qPCR expression values of 1,000 genes (Supplementary Figures 1B, 8). This protocol is also strand-specific (Supplementary Figure 9), unlike most of the previously reported protocols for full-length scRNAseq (for example, the SMART_v4 protocol). Given these observations, we thus decided to further investigate the SMARTer protocol and its adaptability to a higher-throughput platform.

### Adapting SMARTer to a Higher-Throughput, Strand-Specific Total RNA scRNAseq Protocol

To increase the throughput of the SMARTer protocol, we chose to adapt it to an open array platform from Scienion that integrates single-cell isolation with nanoliter reagent dispensing capacity. The instrument’s cellenONE automated single-cell isolation feature uses piezo acoustic technology and optical monitoring of picodroplets to dispense cells: a droplet is dispensed into a waste recovery receptacle if the distal tip of the nozzle is automatically determined to contain no cell or multiple cells, or into a well if a single cell is found in the ejection zone. We adapted the instrument to dispense into a Wafergen chip (Takara) containing 5,184 nanoliter-scale wells, maximizing potential throughput and constraining reagent volumes to nanoliters in a fashion similar to that described previously for the Direct Library Preparation Plus (DLP+) single-cell genome protocol (Laks et al., 2019).

To determine the fidelity of single-cell dispensing, we stained cells and upon imaging of the chip, counted instances of no cell, single cell or multiple cells within individual wells. Based on seven independent runs, three different cell types and a total of 6,216 cells, post-imaging calls of single cells were made for 91–98% of the wells (Supplementary Figure 10). Importantly, all wells with multiple cells could be identified based on the image of unstained cells in the cell dispensing nozzle, and these could thus be excluded from downstream analyses. Given the protocol’s high fidelity in delivering one cell per well, we adopted a staining-free protocol for our scRNAseq application. Modifications to the SMARTer protocol included expansion of the indexing capacity beyond 96 cells and workflow changes to enable early pooling of indexed cells before rRNA depletion and adaptation to our automated system as depicted in Figure 1. We hereafter refer to this method as DLP-scRNAseq.

### Comparison of DLP-scRNAseq With Bulk RNAseq and Orthogonal Assays

To examine the extent to which the DLP-scRNAseq protocol introduced artifacts affecting sequencing data quality or expression dynamics, we compared our single-cell data to data generated from populations of cells using the same protocol but in a 96-well format. Specifically, we compared 92 individually indexed cells and a pool of 402 individual cells with identical index, all of which were processed according to the DLP-scRNAseq protocol, to pools of 10 cells and 500 cells that were processed in bulk. An immortalized normal human astrocyte (NHA) cell line was used for these comparisons (Sonoda et al., 2001).

**FIGURE 2:**
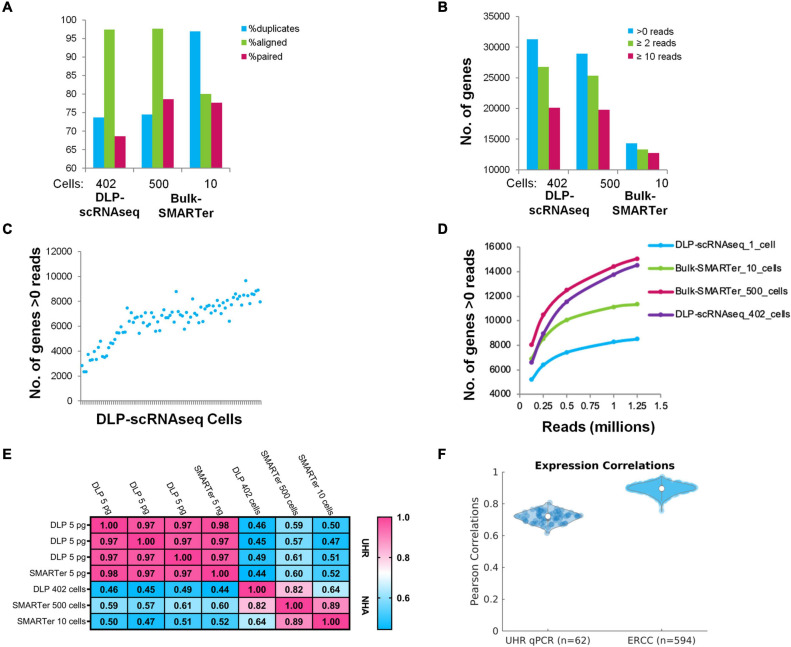
Comparisons of DLP-scRNAseq data and bulk RNAseq data and benchmarking using orthogonally generated data. **(A)** Alignment-based metrics of scRNAseq (DLP-scRNAseq) data vs. bulk (SMARTer) RNAseq data. 80 million reads were used for each data set. **(B)** Number of genes detected in DLP-scRNAseq data vs. bulk RNAseq data. 80 million reads were used. **(C)** Number of genes detected by at least one read in each of the 90 uniquely barcoded single cells (blue dots). Cells are sorted in ascending order based on number of reads. **(D)** Evaluation of sequencing saturation. Reads were down-sampled to numbers between 0.125 and 1.25 million and the number of genes with >0 reads was enumerated at each sampling depth. Curve slopes are indicative of the yield of new genes sampled as a function of sequencing depth, with steeper slopes indicative of lower saturation levels. **(E)** Pearson correlation values comparing expression values from bulk-based RNAseq (SMARTer) data with DLP-scRNAseq data for UHR and NHA data. **(F)** Pearson correlations comparing DLP-scRNAseq and qPCR data (UHR) and known synthetic RNA measurements (ERCC).

Analyses of sequencing quality (Figure 2A) and quantification of the number of genes detected (Figure 2B) indicated data of comparable quality between libraries generated using our DLP-scRNAseq protocol and those generated from bulk cell populations, suggesting that quality and gene detection were preserved as reaction volumes were reduced to nanoliter levels.

Although DLP-scRNAseq libraries from two of the 92 individually indexed cells produced only 462 and 602 reads, respectively, reads from the remaining libraries yielded from 98,222 to 1,773,656 reads with an average of 757,791 reads per cell. The average number of expressed genes detected per cell was 7,371 (+/− 903) (Figure 2C). As shown in Figure 2D, it appears that saturation of the number of genes detected was not reached at 1 million reads per cell.

Gene-level expression analysis showed that data from the DLP-scRNAseq pool of single cells were highly correlated with those of SMARTer libraries from bulk cells (Pearson’s correlation = 0.82) (Figure 2E). We included 5 pg UHR RNA in selected wells to represent the amount of RNA expected from a single cell. The Pearson correlation of gene-level expression from these 5 pg DLP-scRNAseq UHR libraries to bulk SMARTer libraries from 5 ng UHR total RNA input was 0.97–0.98 (Figure 2E), indicating good expression concordance between single-cell and bulk implementations of the method.

We further evaluated the accuracy of gene expression quantification, comparing the single-cell protocol to public qPCR data for 1,000 UHR genes and considered the expected expression levels of the 92 ERCC spike-in RNAs. The average Pearson correlation between the qPCR data and DLP-scRNAseq data for the 1,000 UHR genes was >0.7 and the average correlation between expected and observed levels of ERCC RNAs was >0.9 (Figure 2F), once again indicating that the DLP-scRNAseq protocol generated accurate gene expression measurements.

To compare the sensitivity, accuracy and technical variability of DLP-scRNAseq, we compared counts of ERCC RNA-aligning reads in our protocol with those from publicly available gold standard full-length SMART-seq single-cell data (PRJEB20161, PRJEB20163, and PRJEB20166). To measure sensitivity, logistic regression to estimate the concentration at which an ERCC RNA had a 50% likelihood of being detected was applied as described previously (Svensson et al., 2017). The molecular limit of detection was derived from these results.

Based on an equivalent total number of reads (100,000 reads per cell), the median limit of detection with 50% probability was considerably lower for our protocol compared to datasets generated using SMART-seq protocols (50 vs. 268, 133, and 216) (Supplementary Figure 11A). Pearson’s correlation values of expected vs. observed ERCC RNA levels also indicated that our protocol was more accurate (median *R* = 0.84) than the SMART-seq protocols (median *R* = 0.68, 0.58, and 0.70, respectively) (Supplementary Figure 11B). Sequencing depth had a negligible effect on the correlation values (Supplementary Figure 12). Variability of ERCC expression, based on normalized total read numbers (100,000 total reads per cell), was assessed by: (1) adjusting the number of cells based on the sample with the fewest cells (random sampling of cells was applied to match the minimum number); (2) removing ERCCs with 0 reads in all cells within a sample; (3) calculating average expression levels of each of the ERCC RNAs across all cells within a sample; and (4) computing the coefficient of variation (% CV) for each ERCC RNA (standard deviation divided by the average expression level across cells within a sample). As shown in Supplementary Figure 13, % CV was comparable between the different protocols.

### DLP-scRNAseq Can Yield Sequences Spanning Entire Transcripts

**FIGURE 3:**
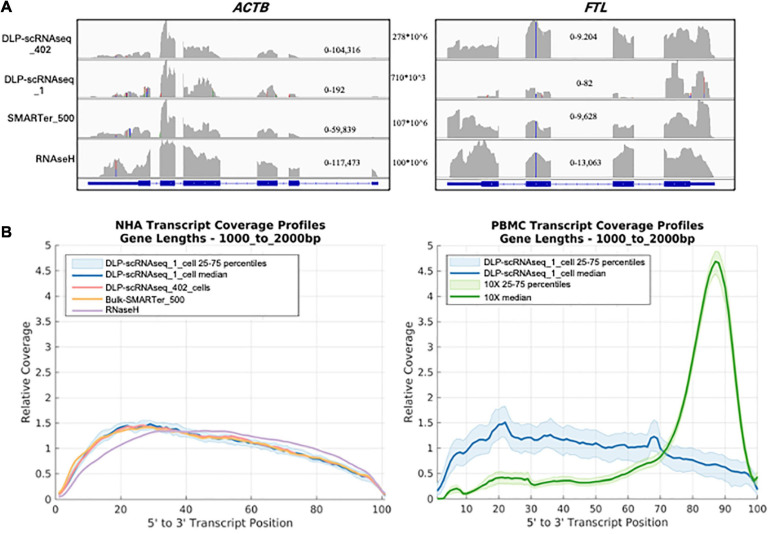
DLP-scRNAseq profiles full-length RNAs **(A)** A screen shot of an Integrative Genomics Viewer image of the genomic region spanning the *ACTB* (left) and *FTL* (right) genes. DLP-scRNAseq _1 is a single-cell library with a read number (710,000) representative of that obtained for other single cells (mean = 757,791 reads). Genomic location-specific read depth ranges are indicated within each plot, and the total number of reads for each library is shown between the plots. **(B)** Comparison of the normalized coverage of transcript bodies, from 5′ (left) to 3′ (right) of all annotated termini (3′ being the location of the polyadenylation site), achieved using DLP-scRNAseq and bulk RNAseq data. The left panel shows data from NHA cells and the right panel shows data that were generated from PBMCs. For the PBMC plot, data that were generated using the 3′-end profiling 10× Chromium protocol are also shown, illustrating the 3′ end bias expected from the 10X platform.

**FIGURE 4:**
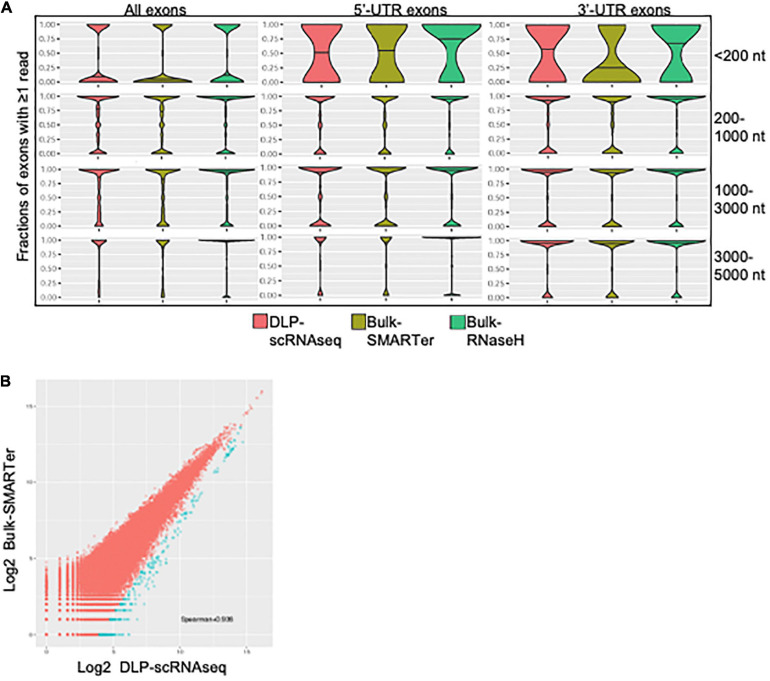
Exon level quantification of gene expression. **(A)** Comparisons of sensitivity of exon-level detection between DLP-scRNAseq and bulk protocols. Violin plots show the distributions of the density of the data representing various fraction of exons covered by one or more reads (*Y*-axis) for various ranges of transcript lengths in Ensembl annotations. Shown are data for all exonic regions (left panel), for full and partial exons falling within 5′ untranslated regions (UTRs) of transcripts (middle panel), and for full and partial exons falling within 3′ UTRs of transcripts (right panel). The coverage across coding regions of transcripts ranging in length from 200 to 5,000 nucleotides (178,348 transcripts in total) was similar between data from the DLP-scRNAseq pool of single cells and bulk libraries generated using SMARTer and RNaseH methods. Transcripts that are shorter than 200 nt (9,750 in total) showed more variable coverage, particularly at the 3′- and 5′-UTR regions. **(B)** A log-log plot of exon-level expression values comparing DLP-scRNAseq to bulk SMARTer data. Correlation values were calculated for exons with one or more reads in both datasets. The Spearman correlation was 0.93, indicating high similarity of expression of 333,517 exons. Exons captured to a higher extent with DLP-scRNAseq than SMARTer (∼459 exons, blue dots), falling below the diagonal (using the formula y–1.28× < –5), spanned all chromosomes and mapped to 354 genes.

Several lines of evidence supported the notion that our DLP-scRNAseq protocol could recover sequences spanning entire transcripts and not only terminal transcript regions. First, visual inspection of randomly selected highly expressed genes, such as the *ACTB* and *FTL* genes in Figure 3A, showed that sequence reads mapped to all annotated exons. Second, the distribution of sequence reads along the 5′–3′positions of transcript bodies was comparable between libraries that were generated from single cells and bulk populations of cells, including those that were generated using the standard rRNA depletion protocol (RNaseH) (Figure 3B). Third, exon-level expression analysis revealed that the fractions of exons that were covered with at least one read were comparable between a pool of single cell libraries (*n* = 402 cells) and bulk RNAseq libraries regardless of transcript length and the 5′ or 3′ location of the exons (Figure 4A). The exon-level expression from the pool of single-cell libraries was highly correlated with that of a bulk RNAseq library from 500 cells (Spearman correlation = 0.936) for the 333,517 exons detected in both the pool of single cell libraries and the bulk library (59% of the 562,205 total exons in the Ensembl annotation) (Figure 4B). In addition to the commonly detected exons, 47,904 exons (from 17,704 genes) were uniquely detected in the pool of single cells and 21,429 exons (from 10,534 genes) were uniquely detected in the bulk library. The average RPKMs of the uniquely detected exons were 0.646 and 0.762 for the single-cell pool and the bulk libraries, respectively. The expression level of these uniquely detected exons was ∼27-fold lower compared to the average RPKM of the exons detected in both the single-cell and bulk libraries (RPKMs of 18 and 20, respectively), indicating that highly expressed genes were detected more consistently, while the detection of less abundantly expressed genes was less robust, regardless of the method used.

**FIGURE 5:**
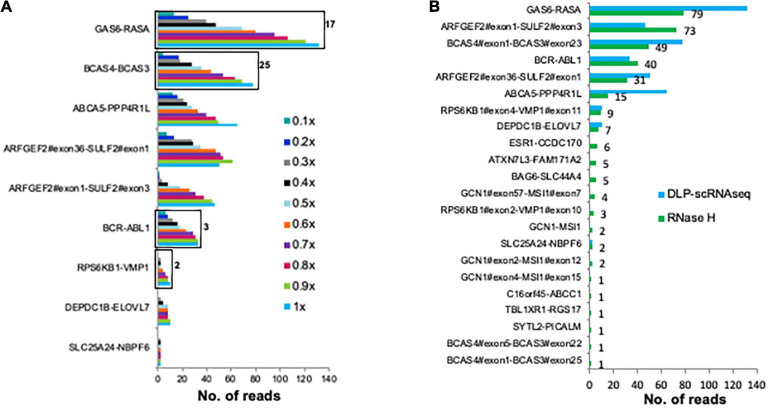
DLP-scRNAseq can be used to detect fusion transcripts. **(A)** Reads from 62 UHR (5 pg total RNA) libraries that were generated using the DLP-scRNAseq protocol were pooled and analyzed for intergenic transcript fusion junctions, previously identified and validated using qPCR (Sakarya et al., 2012). Black boxes indicate events that were confirmed by *de-novo* transcript sequence assembly (Nip et al., 2019). The number on the black boxes indicate the number of contiguous reads covering the fusion transcript. The fraction of down-sampled reads is indicated in the legend (e.g., 1× corresponds to 250 million reads, 0.1× corresponds to 25 million reads). The fewest total reads corresponds to 0.4 million/cell and the highest total number of reads represents 4 million reads per cell. **(B)** Comparison of the sensitivity of detection of fusion transcripts between the pool of UHR libraries that were generated using DLP-scRNAseq data and data from UHR bulk libraries (100 ng total RNA) that were generated using the RNaseH protocol. The number on the black boxes indicate frequencies of detection for each fusion event.

Finally, we assessed whether fusion transcripts could be detected in our data. For this analysis, we made use of previously identified UHR fusion transcripts (Sakarya et al., 2012; Figure 5A). Twenty-two of these fusion transcripts were detected in UHR libraries that were generated using the bulk RNaseH protocol; of these, nine were detected using DLP-scRNAseq (Figure 5B). The fusion events that were not detected in the DLP-scRNAseq data were of low abundance, as they were detected in the bulk data with fewer spanning reads compared to the rest of the fusion events (Figure 5B). These data indicated that DLP-scRNAseq can capture reads that span entire transcripts, depending on the abundance of such transcripts and sequencing depth.

### DLP-scRNAseq Allows for the Profiling of Non-polyadenylated Transcripts

**FIGURE 6:**
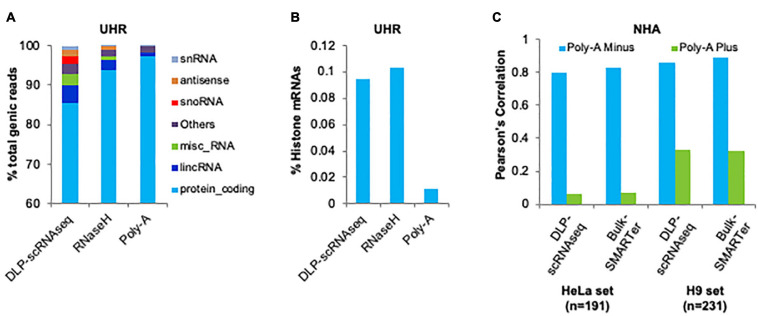
Demonstration of the capacity of DLP-scRNAseq to capture both polyadenylated and non-polyadenylated RNAs. **(A)** Detection of various RNA biotypes. The proportion of various classes of detected transcripts is shown for a pool of single cell libraries generated using DLP-scRNAseq and for bulk libraries that were generated using the SMARTer and RNaseH protocols. Total UHR RNA was used as input. **(B)** Detection of histone mRNAs. The proportion of histone transcripts is shown for a pool of single cell libraries generated using DLP-scRNAseq and for bulk libraries that were generated using the SMARTer and RNaseH protocols. Total UHR RNA was used as input. **(C)** Detection and quantification of polyA^–^ RNAs in scRNAseq data and bulk RNAseq data from NHA cells. Pearson correlations between expression profiles generated by DLP-scRNAseq or SMARTer and expression values of genes whose expression was enriched in polyA^–^ and polyA^+^ fractions are shown.

**FIGURE 7:**
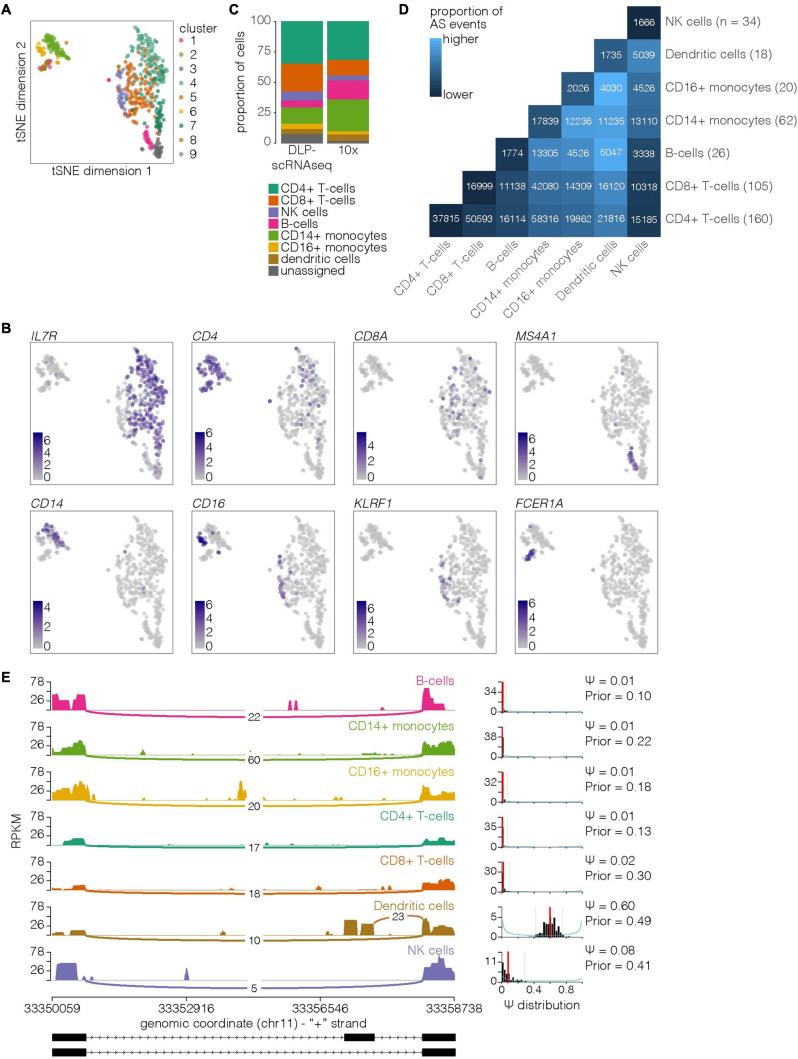
Classification of PBMC cell types based on expression profiles that were generated using DLP-scRNAseq. **(A)** tSNE plot with DLP-scRNAseq cells colored by cluster. **(B)** tSNE plots with cells colored by normalized expression of the indicated marker gene. **(C)** Proportions of cells identified as the indicated cell types in the DLP-scRNAseq and 10X PBMC datasets. **(D)** Heatmap showing proportion of all possible event pairs that were found to be alternatively spliced between indicated cell types. The number of cells assigned to each cell type is indicated on the right: the total number of possible event pairs was calculated by (# of cell type 1 cells × # of cell type 2 cells × total number of transcripts tested). Absolute numbers of AS events between cell type pairs are also shown on the heatmap. **(E)** Example of a cell type-specific AS event (*HIPK3*, BRIE transcript ID ENSG00000110422.7.AS2). Left: sashimi plots showing read densities (in RPKM) within pools of cells assigned to the same cell type. Junction reads linking exons are also indicated with lines and labeled by their count. The outside exons are exons 3 (left) and 4 (right) in most Gencode v19 *HIPK3* transcripts (16 exons total in ENST00000525975.1, ENST00000379016.3, and ENST00000456517.1; 17 exons total in ENST00000303296.4); the middle exon, which is more frequently retained in dendritic cells compared to the other cell types shown, is specific to transcript ENST00000534262.1 (exon 2 of 4). Right: posterior distributions (blue curve, histogram in black) learned by BRIE for each cell type. Red bar depicts the mean, and the 95% confidence interval is indicated by dashed lines. The posterior (Ψ) is a measure of the frequency of exon inclusion (0–never; 1–always).

We evaluated the capacity of DLP-scRNAseq to profile diverse species of RNA, including those lacking polyA+ tails. We merged the sequence reads from 62 UHR libraries that were each generated from 10 pg total RNA using DLP-scRNAseq and compared the resulting proportion of RNA biotypes to those that were detected in libraries that were generated from 10 to 25 ng total UHR RNA using rRNA depletion (RNaseH) and polyA-enriched protocols, respectively. In the DLP-scRNAseq data, 85.6% of the reads were mapped to protein-coding genes and 4.3% of the reads were mapped to long intergenic non-coding RNAs (lincRNAs) (Figure 7A). In the RNaseH-derived data, 93.8% of total number reads were mapped to protein-coding genes and 2.6% of total number reads were mapped to lincRNAs (Figure 6A). In the data obtained using the polyA-enriched protocol, 97.5% of total number reads were mapped to protein-coding genes and only 0.95% of total number reads were mapped to lincRNAs (Figure 6A). There was also a higher proportion (4.7%) of other non-coding RNAs such as antisense RNAs, small nuclear RNAs (snRNAs) and small nucleolar RNAs (snoRNAs) in the DLP-scRNAseq data compared to the RNaseH (0.98%) and polyA data (0.24%) (Figure 6A), which is consistent with the notion that our protocol can be used to profile a range of RNA biotypes.

Non-coding RNAs, including lincRNAs, may be polyadenylated (Ravasi et al., 2006) while histone mRNAs are among those lacking polyA tails (Marzluff et al., 2008). We evaluated the proportion of histone mRNAs in the UHR DLP-scRNAseq, RNaseH and polyA-enriched libraries described above. There were eight and ninefold enrichments of histone mRNAs in the DLP-scRNAseq and RNaseH libraries, respectively, compared to the polyA-enriched library (Figure 6B), indicating that these protocols effectively capture histone transcripts lacking polyA tails.

Yang et al. (2011) previously identified 278–324 transcripts that were enriched in polyA^–^ fractions in two different human cell lines. Approximately 95% of these transcripts were detected in our DLP-scRNAseq data from 402 pooled NHA cells. We compared the gene-level expression of these transcripts in our data from pooled NHA cells to those that were reported from the polyA^–^ fraction in Yang et al. (2011) and found the Pearson expression correlations to be 0.80 and 0.86 when compared to the values from the two cell lines; Figure 6C). The corresponding values for the bulk SMARTer protocol were 0.83 and 0.89, respectively. In contrast, Pearson correlations using values from the polyA^+^ fraction were lower (0.06 and 0.33 for DLP-scRNAseq and 0.07 and 0.32 for bulk SMARTer; Figure 6C), which likely reflects background noise consistent with the transcripts being not polyadenylated.

Next, we examined whether DLP-scRNA could detect enhancer RNAs (eRNAs), which represent a class of non-polyadenylated nuclear RNAs (Lam et al., 2014). To do so, we used a previously described approach (Hayashi et al., 2018) that leveraged genomic coordinates from the GENCODE and CAGE FANTOM databases. First, we performed comparative analysis of eRNAs for the single-cell protocols described above, namely SMARTer, SMART_V4, exome, RNaseH (RBD), and modified RBD, using 10 ng UHR total RNA input and a normalized number of total reads (10 million). As expected, the exome approach resulted in negligible levels of eRNAs and the polyA-based SMART_V4 similarly showed minimal eRNA levels (Supplementary Figure 14A). The SMARTer protocol, which underpins our single cell protocol, displayed the highest sensitivity of eRNA detection at a level comparable to that of the modified rRNA depletion protocol (Supplementary Figure 14A). Using a comparable number of NHA cells and normalized number of total reads (80 million), the pooled data generated using our DLP-scRNAseq protocol showed a comparable level of eRNA detection relative to that of the bulk SMARTer protocol (Supplementary Figure 14B).

Circular RNAs (cRNAs) are another class of non-polyadenylated RNAs. Using a recently reported approach (Zhang et al., 2020), we compared the protocols described above using varying input amounts (0.1–10 ng) of UHR total RNA. This analysis showed that the SMARTer protocol displayed > 4-fold higher cRNA levels compared to the other protocols. The exome and SMART_4 approaches resulted in the lowest cRNA recovery (Supplementary Figure 15A). Supplementary Figure 15B shows that DLP-scRNAseq identified ∼50% of the cRNAs that were detected using the bulk SMARTer protocol from a comparable number of NHA cells. Taken together, these data indicate that DLP-scRNAseq can be used to profile both polyA^+^ and polyA^–^ transcripts.

### DLP-scRNAseq Enables Cell Type Classification Using a Biologically Heterogeneous Sample

To assess the capacity of the DLP-scRNAseq protocol to discern cell types from a biologically complex sample, we processed cryopreserved human peripheral blood mononuclear cells (PBMCs) using DLP-scRNAseq. Of the libraries from 518 cells that were sequenced on one-third of a HiSeq 2500 lane (188 million reads), 473 libraries had > 100,000 reads with an average of 383,812 reads per cell. The average number of genes detected per cell was 2,830 (Supplementary Figure 16).

To identify distinct cell types, we first performed clustering analysis on the expression profile of the PBMCs, identifying nine clusters (Figure 7A). Examination of genes that marked the expression of each cluster (Supplementary Table 2) revealed the anticipated cell types at expected ratios (Kleiveland, 2015), namely T cells [clusters 4, 5, and 7 (∼57%)], collectively marked by expression of *IL7R* (Carrette and Surh, 2012) and the T cell surface glycoproteins *CD5* and *CD6* (Gonçalves et al., 2018); B cells [cluster 1 (∼6%)], enriched for expression of the B cell receptor signaling molecule *MS4A1* (Polyak et al., 2008); CD14+ (cluster 2) and CD16+ (cluster 6) monocytes (Ziegler-Heitbrock et al., 2010) (∼18%); natural killer cells [cluster 9 (∼7%)], enriched for markers such as *KLRF1*(Moretta et al., 2003) and *KLRD1*(Borrego et al., 2005); and dendritic cells [cluster 8 (∼4%)], marked by high expression of *CD74* and *FCER1A* (Greer et al., 2014; Figure 7B). Within the T cell clusters, cells in clusters 4 and 7 (∼60% of T cells) expressed *CD4*, whereas cluster 5 (∼40% of T cells) was enriched in cells expressing *CD8A*. Cells in cluster 3 were not enriched for cell type-specific markers (FDR < 0.05). However, closer examination of QC measures revealed that this population had a high proportion of reads aligned to ERCCs (Supplementary Figure 17A), indicating that these may have been poorer quality libraries that were not filtered using standard QC methods.

To analyze a comparable dataset produced using a different platform, we also obtained data from 1,223 PBMCs profiled using the 10X Genomics Chromium platform^6^. Our clustering analysis also identified nine clusters for this dataset which displayed similar expression patterns to those found in the DLP-scRNAseq dataset: cell clusters 2, 5, and 6 (44% of cells) expressed markers of T cells such as *IL7R*, cluster 4 (∼16%) was enriched for expression of the B cell marker *MS4A1*, cells in cluster 9 expressed the NK cell marker *KLF1*, clusters 3 (∼26%) and 7 (∼3%) appeared to be composed of CD14^+^ and CD16^+^ monocytes, respectively, and cells in cluster 1 (∼5%) displayed high expression of *CD74* and *FCER1A*, indicating that they were likely dendritic cells (Supplementary Figures 17B,C and Supplementary Table 2). Similar to our observations for the PBMC dataset obtained using DLP-scRNAseq, cells in cluster 8 were not characterized by a pattern of marker gene expression that was clearly indicative of a cell type, and this cluster appeared to be composed of lower-quality cells as evidenced by its high proportion of read counts assigned to mitochondrial genes (Supplementary Figure 17D). Overall, the cell type proportions identified in the DLP-scRNAseq and 10X datasets were comparable (Figure 7C) despite some differences that can also be attributed to the individual source variation of the PBMC samples.

To determine whether DLP-scRNAseq data could be used to identify alternatively spliced (AS) transcripts, we used BRIE (Huang and Sanguinetti, 2017) to quantify exon inclusion events. We first performed pairwise comparisons between individual cells and, for each pair of cell types, calculated the proportion of all possible events that were identified as alternatively spliced (Bayes factor ≥10; Methods). Pairs of cells assigned to the same cell type consistently had a lower proportion of AS events between them than pairs of cells assigned to different cell types (Figure 7D). Additionally, pairs of cells from similar cell types (e.g., CD4+ T-cells and CD8+ T-cells) tended to have lower proportions of AS events between them than pairs of cells assigned to more distinct cell types (e.g., B-cells and dendritic cells). These results both supported the clustering-based cell type assignments and indicated that alternative splicing events can be identified between individual cells at ratios that are consistent with expected cell type differences.

We next performed alternative splicing analyses comparing distinct cell types (Methods). We identified 3,008 AS events between at least two cell types (Bayes factor ≥10), and from this list identified 179 cell type-specific events (example shown in Figure 7E; full results in Supplementary Table 3). Notably, these included events that have previously been identified: for example, *BTG3*, which has been found to be differentially spliced in lung cancers (Chen et al., 2013), appeared to be most highly expressed in T-cells and NK cells, and inclusion of exon 4 was significantly higher in CD4+ T-cells than other cell types (Supplementary Figure 18A). Similarly, several *CTSB* splice variants, including one lacking exon 2, have been shown to be differentially expressed in cancer (Liyanage et al., 2019), and we found evidence in our dataset that CD14+ monocytes had significantly more expression of exon 2 than other cell types (Supplementary Figure 18B). Our results thus indicate that DLP-scRNAseq can be used to study AS transcripts enriched in comparisons of cell types.

Verboom et al. (2019) recently reported single-cell profiling results from the same scRNAseq (SMARTer) kit that we used here. Another study also reported on a similar protocol (Isakova et al., 2020). Unique contributions of our work here include: analyses revealing the ability of DLP-scRNAseq to discern cellular heterogeneity; the orthogonal validation of expression accuracy using qPCR on 1,000 genes; our comparisons to bulk total RNAseq data; and expanded analysis of full-length transcript coverage. Further, our work adapts the kit to a different automation platform of single-cell isolation and library construction that allows for the simultaneous processing of hundreds to thousands of cells, while previous protocols are limited to 96 cells per run. Our data demonstrate that our approach allows for measurements of full-length transcript expression of both polyA^+^ and polyA^–^ RNAs at a single-cell resolution for hundreds to thousands of cells per run, thus providing an avenue to comprehensively study gene expression in the context of complex, heterogeneous biological samples at single-cell resolution.

## Data Availability Statement

The datasets presented in this study can be found in the NCBI Sequence Read Archive (accession SRP286135).

## Author Contributions

SH and MAM: conception. SH, RDC, VGL, LW, and MAM: writing. SH, RDC, VGL, LW, and KB: graphics. SH, RDC, VGL, LW, SP, SB, KN, and DLT: execution. SH, MB, EC, RJNC, RAM, AJM, KLM, YZ, MH, SA, SJMJ, and MAM: grant application and supervision. All authors contributed to the article and approved the submitted version.

## Conflict of Interest

The authors declare that the research was conducted in the absence of any commercial or financial relationships that could be construed as a potential conflict of interest.

## Footnotes

1 https://support.10xgenomics.com/single-cell-gene-expression/datasets/3.0.0/pbmc_1k_v3

2 http://cf.10xgenomics.com/supp/cell-exp/refdata-cellranger-hg19-3.0.0.tar.gz

3 https://sourceforge.net/projects/brie-rna/files/annotation/

4 https://github.com/huangyh09/briekit

5 https://github.com/lh3/seqtk

6 https://support.10xgenomics.com/single-cell-gene-expression/datasets/3.0.0/pbmc_1k_v3
