# Supplementary material for: A Scalable Strand-Specific Protocol Enabling Full-Length Total RNA Sequencing From Single Cells
Source: Front Genet. 2021 Jun 3;12:665888. doi: 10.3389/fgene.2021.665888 (PMC8209500; doi:10.3389/fgene.2021.665888)
Supplement: Supplementary file 4 [file Table_4.DOCX]

**A scalable strand-specific protocol enabling full-length total RNA sequencing from single cells**

Simon Haile^1^, Richard D. Corbett^1^, Veronique LeBlanc^1^, Lisa Wei^1^, Stephen Pleasance^1^, Steve Bilobram^1^, Ka Ming Nip^1^, Kirstin Brown^1^, Eva Trinh^1^, Jillian Smith^1^, Diane Trinh^1^, Miruna Bala ^1^, Eric Chuah^1^, Robin J. Coope^1^, Richard A. Moore^1^, Andrew J. Mungall^1^, Karen Mungall^1^, Yongjun Zhao^1^, Martin Hirst^1^, Samuel **Aparicio**^2^, Inanc Birol^1,3^, Steven J. Jones^1,3^ and *Marco A. Marra^1,3^

^1^Canada’s Michael Smith Genome Sciences Centre, BC Cancer, Vancouver, British Columbia, Canada

^2^Department of Molecular Oncology, BC Cancer, Vancouver, British Columbia, Canada

^3^Department of Medical Genetics, University of British Columbia, Vancouver, British Columbia, Canada

*Corresponding author:

675 West 10^th^ Avenue, Vancouver, BC, Canada V5Z 1L3

Tel: 1-604–875–8168; Fax: 1-604-675-8178; Email: [mmarra@bcgsc.ca](mailto:mmarra@bcgsc.ca)

This document contains supplementary information on the following aspects of the paper specified above:

1. Supplemetary Figure Legends
2. Supplementary Methods and Materials
3. Cost estimate breakdown

**SUPPLEMENARY FIGURE LEGENDS**

**Supplementary Figure 1.** Evaluation of scRNAseq protocols. (A) Features of the protocols that were considered in this study. (B) Summary of correlation data from inter-protocol comparisons. Data are representative of the average of the total RNA input amounts used. The first column (nL automation) indicates the possibility of performing the protocol in one reaction vessel without purification in between the various steps (+ or -) and the degree of the simplicity of the protocol in an ascending order (+, ++ or +++). The next four columns are measures of the extent of correlation of expression (R) of the RNAseq data generated using the various protocols compared to ERCC spike-in expected values, measurements obtained using standard rRNA depletion protocol (RNaseH), measurements obtained using poly(A)-based protocol, and previous qPCR measurements, respectively. The last four columns are relative values where values obtained using the various protocols are described as a fraction of the corresponding values obtained using the SMART_V4 protocol. “Diversity” refers to the number of genes detected with >0 reads. “Filtered reads” excludes reads that did not align to the human genome and those that are of rRNA or mitochondrial genome origin.

**Supplementary Fig 2.** Comparison of the alignment tools, JAGuaR and STAR with regard to the number of genes detected (upper panel) and the percentage of alignment of reads (lower panel).

**Supplementary Fig 3-5.** Comparison of post-alignment metrics between the candidate scRNA-seq protocols.

**Supplementary Fig 6.** Positional profile of base errors.

**Supplementary Fig 7.** Expression correlations between the candidate scRNA-seq protocols. Upper panel is a heatmap of Pearson’s correlation matrix based on thousands of genes expressed in UHR. Lower panel is based on correlations of observed vs expected ERCC spike-in RNAs.

**Supplementary Fig 8.** Orthogonal validation of expression accuracy. Previous qPCR data for ~1000 mRNAs were compared with RNA-seq data generated using the various candidate scRNA-seq protocols.

**Supplementary Fig 9.** Strand-specificity. (A) Comparison of exon coverage and strand-specificity between the candidate scRNA-seq protocols. A screen shot of an Integrative Genomics Viewer image of the genomic region spanning the c-MYC gene. Red and Blue colours designate strand-specific reads. (B) Comparison of strand-specificity between the single cell protocol (DLP-scRNAseq) and rRNA depletion-based protocol (RNaseH) using UHR as an input.

**Supplementary Fig 10.** Fidelity of CellenONE single cell dispensing.

**Supplementary Fig 11.** Comparison of sensitivity (A) and accuracy (B) of DLP-scRNAseq with SMART-seq protocols using ERCC spike-in RNAs. The number of cells profiled in each experiment is indicated in the top violin plots.

**Supplementary Fig 12.** ERRC read depth relationship with accuracy for DLP-scRNAseq and the comparator SMART-seq protocols.

**Supplementary Fig 13.** Comparison of technical variability of DLP-scRNAseq with SMART-seq protocols using ERCC spike-in RNAs. The coefficient of variation (CV) across cells for each of the ERCC spike-in RNAs is shown.

**Supplementary Fig 14.** Comparison of enhancer RNA detection between SMARTer and other protocols using UHR input amount (A) and between DLP-scRNAseq and bulk SMARTer protocols (B). The DLP-scRNAseq data is pooled from 402 cells and the bulk SMARTer data is from 500 cells.

**Supplementary Fig 15.** Comparison of circular RNA detection between SMARTer and other protocols using UHR input amount (A) and between DLP-scRNAseq and bulk SMARTer protocols (B). The DLP-scRNAseq data is pooled from 402 cells and the bulk SMARTer data is from 500 cells.

**Supplementary Fig 16.** Gene diversity for each of the uniquely barcoded PBMC single cells. The numbers of genes with > 0 reads are shown. X-axis values are sorted in ascending order based on number of reads.

**Supplementary Fig 17**  A. Violin plots depicting the distribution of percent of reads assigned to ERCCs across clusters in the DLP-scRNAseq data. B. tSNE plot with 10X cells coloured by cluster. C. tSNE plots with cells coloured by normalized expression of the indicated gene. D. Violin plots depicting the distribution of percent of reads from mitochondrial genes across clusters in the 10X data.

**Supplementary Fig 18.**  Examples of cell type-specific splicing events. Sashimi plots showing cell type-specific splicing patterns for *BTG3* (A) and *CTSB* (B). Left: sashimi plots showing read densities (in RPKM) within pools of cells assigned to the same cell type. Junction reads are also indicated with lines and labeled by their count. Right: prior distributions (blue curve, histogram in black) learned by BRIE for each cell type. Red bar depicts the mean, and the 95% confidence interval is indicated by dashed lines. The posterior (Ѱ) is a measure of the frequency of exon inclusion (0 - never; 1 - always).

SUPPLEMENTARY METHODS AND MATERIALS

Single cell spotting and total RNA-seq library construction (DLP-scRNAseq) on the sciFLEXARRAYER S3

# Materials and Equipment

| Name | Supplier | Number | Model or Catalogue # | |
| --- | --- | --- | --- | --- |
| 0.5 mL RNase-free microfuge tube | Ambion | AM12300 |  |  |
| 1.5 mL RNase-free microfuge tube | Ambion | AM12400 |  |  |
| 10% Tween 20 Solution | Bio-Rad | 161-0781 |  |  |
| 21' Bouffant cap, white | VWR | 89107-768 |  |  |
| 500 mL vacuum filters | EMD Millipore | SCGPT10RE |  |  |
| BioRad T100 Thermal Cycler, adapted for Wafergen SmartChips | Wafergen | T100 |  |  |
| Centrifuge, benchtop | VWR | 5810R |  |  |
| Centrifuge-Mini | VWR | Galaxy Ministar |  |  |
| Chip immersion clamp | In House | N/A | N/A | N/A |
| Degassed, filtered DI water | In House | N/A | N/A | N/A |
| Diamond Filter tips DF1000 | Mandel Scientific | GF-F171703 |  |  |
| Diamond Filter tips DF200 | Mandel Scientific | GF-F171503 |  |  |
| Diamond Filter tips DF30 | Mandel Scientific | GF-F171303 |  |  |
| Diamond Filter tips DFL10 | Mandel Scientific | GF-F171203 |  |  |
| Disposable Scalpels | Fisher | 12460451 |  |  |
| Fisherbrand Textured Nitrile gloves – various sizes | Fisher | 270-058-53 |  |  |
| Funnels, Wafergen Collection Kit | Wafergen | P/N 640048 |  |  |
| Gilson P10 pipetman | Mandel | GF-44802 |  |  |
| Gilson P1000 pipetman | Mandel | GF-23602 |  |  |
| Gilson P2 pipetman | Mandel | GF-44801 |  |  |
| Gilson P20 pipetman | Mandel | GF23600 |  |  |
| Gilson P200 pipetman | Mandel | GF-23601 |  |  |
| Glycerol | Sigma | G5516-1L |  |  |
| Gowns- Large (small) | DuPont | 17-990-522(520) |  |  |
| Heat Seals, Seq-Ready TE FLEX Loading Kit | TaKaRa | 640117 |  |  |
| Ice bucket | Fisher | 11-676-36 |  |  |
| Lint free cloth wipes (large) | AlphaWipe | 18-322 |  |  |
| Lint free cloth wipes (small) | AlphaWipe | 18-320 |  |  |
| Lint free stick wipes | Texwipe | 18-383 |  |  |
| Liquid Dispenser-sciFLEX ARRAYER | Scienion | S3 |  |  |
| Microseal® 'A' PCR Plate and PCR Tube Sealing Film | Bio-Rad | MSA5001 |  |  |
| SMARTer® Stranded Total RNAseq Kit v2 - Pico Input Mammalian | Takara Bio Inc. | 634413 |  |  |
| Piezo Dispense Capillary 70 Type 4 | Scienion | P-2002 |  |  |
| Pluronic F127 | Sigma | P2443-250G |  |  |
| Protease | Qiagen | 19155 |  |  |
| Sciclean 8 | Scienion | C-5283 |  |  |
| UltraPure Distilled Water | Invitrogen | 10977-023 |  |  |
| Ultrasonic bath | Fisher Scientific | FS110 |  |  |
| Vacuum pump | Thermo Fisher | GP110 |  |  |
| Water Bath, Isotemp GPD 02 | Fisher Scientific | FSGPD02 |  |  |

# Procedure

## Note: For detailed instructions for use of the sciFLEXARRAYER S3 (“spotter”) and/or SmartChips, refer to LIBPR.0168 (Spotting SmartChips with the Scienion sciFLEXARRAYER S3). For instructions on creating field files and other data tasks, see LIBPR.0170 (Data Processing for SmartChip Single Cell Experiments).

**IN ADVANCE:**

## Calculator Setup and Field File Generation

- 1. Enter the run ID, cell type(s) and total number of wells in the experiment into the input boxes at the top of the Single Cell RNA Calculator.
  2. For *each reagent* to be dispensed:
     1. Determine which wells will be spotted. Typically spotting schemes would be:
        1. Cell lysis solution spotted to all wells (experimental plus all control wells)
        2. RNA spotted to designated positive control wells
        3. Lysis/fragmentation, reverse transcription and indexing PCR mixes spotted to all wells.
     2. If a reagent is being dispensed to only a subset of wells, enter the number of wells into the input box at the top of the recipe for that reagent.
     3. Check that the total required reagent volume is correctly split into an appropriate number of aspirates with appropriate volumes, up to a maximum of 30 uL. If desired, the calculated number of aspirates for a reagent can be manually overwritten.
     4. Generate field files for reagent dispensing to appropriate subsets of the target wells for each aspirate. For example, if two aspirates are required, you could generate field files for the left and right halves of the target wells.
  3. Once all reagent dispenses have been planned and field files created, save a copy of the calculator to be used in the experiment.

**DAY 1:**

1. **Spotter Setup**

Turn on and set up the spotter, using the instructions for general spotting in the spotter work instruction. If continuing from cell spotting on the same day, omit steps 2.5 through 2.8.

- 1. Turn on the spotter power bar.
  2. Load the standard version of the Scienion AG software, with user **_SCI**.
  3. Set the run directory to ***Desktop\delete_me***.
  4. Set the chiller bath temperature at dewpoint -1°C or -2°C to avoid evaporation. If dewpoint is less than 5°C, set humidify to 37% and a fixed temperature 2-3°C below the minimum dewpoint observed as the humidifier operates.
  5. Prepare 450 mL of degassed filtered DI water for system liquid.
  6. Empty the freshwater carboy and refill with DI water to at least 10 cm deep.
  7. Empty the waste carboy and flush bottle.
  8. Prepare 2 mL of 1% sciCLEAN 8 in wash tray 1 (small).
  9. Mount and prime a PDC 70 Type 4 nozzle, and ensure you have a stable drop with water before proceeding.
  10. Ensure a 0.5 mL collection tube is present under the nozzle camera.
  11. Place your SmartChip on the chiller array. The rear left position (#17) minimizes travel distance between the chip and the camera.

## Cell Lysis and RNA fragmentation pre-spotting

- 1. The lysis/fragmentation mix includes the following per well:

|  | **Reagent (nano L)** |
| --- | --- |
| 1:1000 ERCC | 1 |
| 10x lysis buffer | 0.5 |
| 5X 1st strand buffer | 4 |
| TOTAL | 5.5 |

- 1. Spot lysis/fragmentation mix:
     1. Load field file for lysis/fragmentation mix spotting.
     2. Load lysis/fragmentation mix into reagent plate (aspirate volume + at least 3 µL).
     3. Aspirate lysis/fragmentation mix using the appropriate **TakeProbe_##uL** task (aspirate volume should equal total spotting volume + at least 5 µL).
     4. Optimize voltage & pulse, **Set Nozzle Parameters**, and **Autofocus**. Record nozzle ID, voltage and pulse values in the library construction calculator for the run.
     5. If the drop appears off centre or out of the nozzle focal plane, perform the **Nozzle Head Cam Wizard** to optimize drop positioning.
     6. Get **Drop Volume** and enter into calculator to calculate number of drops to spot.
     7. Use “Edit Field Table…” dialog to set number of drops in the active field file.
     8. Start a **SpotRun_Chiller** run on the appropriate target position.
     9. Within 1-2 rows of spotting after FTRP chip registration, and periodically throughout the run, check drop stability and volume:
        1. Click the yellow **Nozzle Setup** button, then the **Camera** button.
        2. Measure drop volume, and check that the drop shape and volume are still acceptable. Modify and set nozzle parameters if desired.
        3. Click **Continue** on the Continue/Cancel prompt to resume the run.
     10. If chip wells were split for multiple aspirates, repeat the above steps to spot additional wells with different field files.
     11. When spotting is complete, execute a **WFS_sciCLEANtray1_WFS** task to clean the nozzle. Note: if nozzle parameters were changed significantly for reagent dispensing, sciCLEAN 8 and/or water may not spot optimally.
     12. Perform run **Wash_NozzleRemoval_SciCleanTray1**.

## Cell Spotting CellenONE

CellenONE is a software add-on for in-nozzle imaging and cell tracking, with only droplets containing a single cell being dispensed into the SmartChip, and the rest to waste.

- 1. Prepare your cells at ~220,000 cells/mL in particle-free PBS.
  2. Set up spotter with the following details/modifications:

| **Setting** | **Spotter setup for CellenONE Spotting** | | **Also check:** |
| --- | --- | --- | --- |
| Software version: | CellenONE |  | Humidifier filled & connected if needed |
| Directory: | Dedicated run folder |  | SafePosition Z=19000 |
| Target type: | SmartChip_ Chiller |  | Probe = Chilled_Armadillo_384_well_plate |
| FTRP check run: | FTRPcheck_ Chiller |  | System liquid attached with sipper down |
| Spotting run: | CellenOne_ _Chiller |  | Waste carboy emptied |
| Chiller temperature: | Dew point -1°C |  | Flush bottle emptied |
| Fresh water level: | ~10 cm |  | Collection tube below camera |
| sciCLEAN 8 wash: | 2mL 1% sciCLEAN 8 in tray 1 |  | Nozzle primed, drop checked & focused |
| Tween wash: | N/A |  | Nozzle head camera wizard run if needed |
| Nozzle: | PDC90 Type 4 |  |  |

- 1. Prepare system for CellenONE setup:
     1. Ensure that you have selected a dedicated run directory, not delete_me.
     2. Turn off the clean room light and place the blackout panels on the front and (optionally) left side of the spotter enclosure. The front panel should have its viewing cutout at top right. *This is important as CellenONE cell tracking is very sensitive to changes in lighting on the nozzle during cell spotting.*
  2. Prepare and load field files for cells and, if desired, no-cell control wells:
     1. Use a 72x72 single-field file rather than multiple fields in order to correctly record the well positions.
     2. For single-cell applications, the number of drops should be set to **1** for all wells.
  3. Place your SmartChip in the chiller array adapter with the shaded corner at back left and the “Wafergen #####” text at the back.
     1. If using the chiller, also make sure the 4x5 array adapter is seated firmly to the front left corner of the array.
     2. Align the chip squarely to the front left corner of its holder and ensure the chip is sitting flat rather than having one edge resting on the lip of its holder.
  4. Perform a chip image recognition check run:
     1. Start run **FTRPcheck_Chiller**, selecting the target position. The spotter will attempt to identify the back left and back right corners of the chip.
     2. If a chip corner image appears with a single red box centered on the top left corner (chiller run) of the chip, proceed to the 4.7 step.
     3. If instead you receive a prompt reporting "Following target(s) will not be produced", click **Continue**, check that the chip is seated tightly and squarely to the front left corner of its holder and flush on the bottom, then restart the image check run.
     4. If chip registration still fails, use the FTRP window to regenerate the corner registration images for the appropriate target type:

SmartChip_Chiller target:

| Ref. # | Corner | X Offset | Y Offset | Image filename |
| --- | --- | --- | --- | --- |
| 1 | Back left | 0 | 0 | *smartchip_chiller_GSC.bmp* |
| 2 | Back right | 38482 | 0 | *smartchip_chiller_GSC-2.bmp* |

- - 1. Repeat the FTRP check run to confirm that the chip is recognized.
  1. Aspirate PBS and cell suspension
     1. Home the spotter using the **“Home”** quick action button on the Nozzle Setup tab, and replace the 0.5 mL waste collection tube with a new 0.5 mL cell collection tube in front of the camera.
     2. Set expected good Voltage [V] and Pulse [µs] parameters for dispensing PBS cell suspension with your mounted nozzle.
     3. Load 15 µL of particle-free PBS into a new well of the 384-well reagent plate, to be used as a spacer between the cells and system liquid. Do task **TakeProbe_10ul**, click the **“Water dip”** quick action button, then the **“Camera”** quick action button, wait 8 seconds, then check for a stable droplet. Adjust nozzle parameters as needed then click **Set Nozzle Parameters**.
     4. If the drop appears off centre or out of the nozzle focal plane, perform the **Nozzle Head Camera Wizard** to optimize drop positioning.
     5. Load 20 µL of freshly-mixed cell suspension into a new well of the 384-well reagent plate, then do task **TakeProbe_15ul**. Click the **“Water dip”** quick action button, then the **“Camera”** quick action button, wait 8 seconds, then confirm you still have a stable droplet. Adjust nozzle parameters as needed then click **Set Nozzle Parameters** then **Autofocus**.

*Note: aspirating 15 µL of well-dispersed cell suspension at 220,000 cells/mL is typically enough to spot ~1500 cells, but the volume can be adjusted if desired.*

- 1. Additional spotter setup for CellenONE dispensing (work fairly quickly to reduce cell settling in the nozzle):
     1. Under Nozzle Setup, set the LED Pulse [µs] to **7**.
     2. Double-check that the nozzle is at the home or camera position.
     3. Under **Robot Setup > Drives**, edit the SafePosition predefined location and set the Z value (not Z Vel.) to match the Z value of the Target location, and click **OK**. This will allow the nozzle to minimize its vertical movement when moving between the camera and the chip and increase efficiency.

***IMPORTANT****: this lower safe position is* ***only*** *safe for nozzle movements between the CellenONE chip cradle, camera wash station, and sciCLEAN wash tray, and* ***MUST*** *be reset before moving the nozzle to any other position, or the nozzle could crash and break. Place a warning note on the screen over top of the Nozzle Setup tab as a reminder to reset the safe position before performing any other operations.*

- 1. CellenONE setup:
     1. Do task **Cell_Monitor** to open the CellenONE window.
     2. Set initial CellenONE settings as follows. *Note: to access settings on the* ***Advance Settings*** *tab, set Show Advance Settings? to* ***Show.***

| **CellenONE Tab** | **Setting** | **Value** |
| --- | --- | --- |
| Main | Particle Geometry Properties | Off |
|  | Run Data File | Off |
|  | Print Run Data File (checkbox) | Off |
|  | Print Run Data File (text) | Chip or library ID |
|  | Min Mapping Cycles | 100 |
|  | Density Threshold | 0.25 to 0.3 |
| Advance Settings | Save Images | Off |
|  | Negative Control | Off |
|  | Max No. of Trials (for timeout) | 200, or as desired |

- - 1. Generate a background image:
       1. In the **Main** tab, click **Test Droplet** repeatedly until the nozzle image shows no cells anywhere in the nozzle. This may take a lot of clicks.
       2. Quickly go to the **Advance Settings** tab and turn Save Images **On**
       3. Quickly go back to the **Main** tab and click **Get Background** to save a background image into your run folder.
       4. Turn Save Images back **Off** so that only images of spotted cells are saved.
       5. If the displayed backgound image shows that cells have settled into the nozzle, repeat the above steps to generate a new background image.
    2. Set cell morphology threshold values:
       1. In the **Main** tab, click **Test Droplet** repeatedly and check that cells are being properly tracked as they move through the nozzle. Each cell or cluster of cells should have a light blue dot overlaid on it, and dots should not be present outside of cells. If tracking is poor, regenerate the background image.


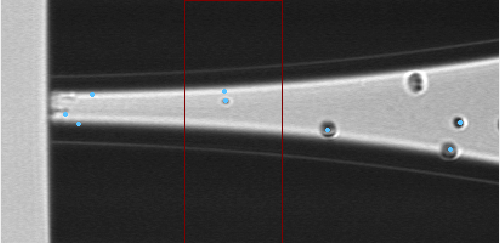


**Figure 1: Example of poor tracking (blue dots outside of cells)**

- - - 1. As you click Test Droplet repeatedly, inspect the Circularity, Elongation and Area properties of the detected cells. Displayed values pertain to the left-most cell being tracked. Determine the maximum circularity (1 = circle, >1 = irregular/corners), maximum elongation (1 = circle, >1 = oval ), and both minimum and maximum area values for typical cells in your suspension
      2. Enter these threshold values in the **Advance Settings** tab to spot only cells within these morphology ranges.
    1. Perform nozzle mapping:

Mapping is used to determine the region of the nozzle that will be expelled in the next droplet by tracking cells during dispensing. If a cell at a given location is still found in the nozzle after the next droplet is dispensed, that location is marked with a dark blue dot, but if it is no longer found, the location is marked green. The location of the *lowermost* blue dot (leftmost in the image) is marked as the upper boundary of the dispense zone. Another boundary line is placed a certain number of pixels above this line (to the right in the image), defining a sedimentation zone to protect against cells sedimenting into the dispense zone between imaging and spotting. When spotting, CellenONE will use these boundaries to identify nozzle contents where one cell is in the dispense zone *and* no cells in the sedimentation zone, then use these for spotting a single cell.


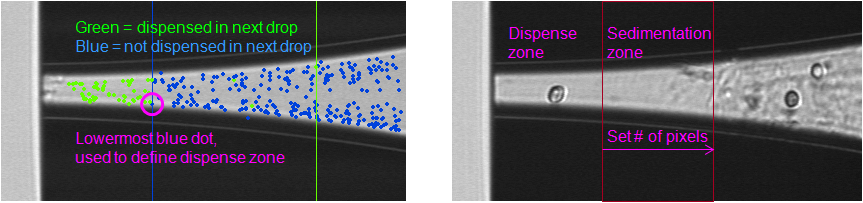


**Figure 2: Examples of successful mapping and nozzle contents selected for single cell spotting**

- - - 1. In the **Main** tab, click **Start Mapping**. Cells will be tracked until both the number of cycles and the total map density reach the specified values.
      2. Once mapping completes, inspect the map image for good mapping. A good map will show good separation between green and blue dots. Uneven dot distribution (Figure 3A) or poor separation of green and blue (Figure 3B) may indicate poor focus or uneven dispensing. Having a few green dots above (right of) the blue dispense zone boundary line is fine, but blue dots located too low will make the dispense zone very small and reduce spotting efficiency.


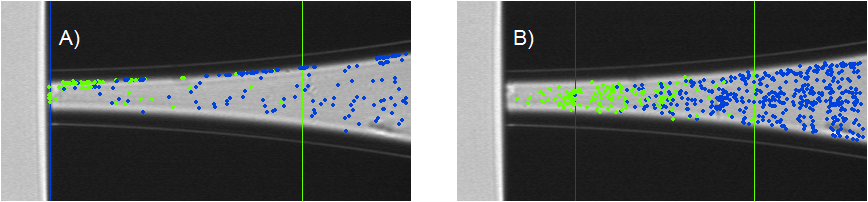


**Figure 3: Examples of poor mapping: A) Uneven distribution and B) Poor blue/green separation**

- - - 1. If mapping is poor, try the following, in this order:
         1. Repeat mapping without changing anything
         2. Go to **Nozzle Setup > Nozzle Offset** and adjust focus. You can use **Autofocus**, or manually adjust the Back/Front step controls and then click **Set Nozzle Parameters**. You will need to get a new background image before repeating mapping.
         3. Adjust nozzle voltage and/or pulse to modify drop dispensing.
         4. Adjust the **LED Pulse [µs]** time. You will need to get a new background image before repeating mapping.
         5. If cells look too concentrated, dilute the cells and re-aspirate.
         6. If cells look clumpy, filter the cell suspension and re-aspirate.
    1. Once you have good mapping, set the Print Run Data File checkbox to **On**. Keep Save Images **Off** to record images of only the spotted cells.
  1. Cell spotting run:
     1. Leave the CellenONE window open, but go back to the main Scienion AG window. You may need to move the CellenONE window aside.
     2. Check that you are using the correct field setup for cells, with **1** drop per well.
     3. Start run **CellenOne_Chiller**, selecting the target position. The run will spot at up to 10 cells per minute, but speed will depend on cell density, clumpiness, and calibration of cell morphology parameters.
        1. If you receive a message saying that the target will not be produced, click **Abort**, *NOT Continue*, as continuing a CellenONE run without target recognition will cause the software to stall. Click **OK**, check chip placement and/or repeat reference image generation, then restart the run.
     4. Monitor the run for the first little while, and periodically thereafter.
        1. If CellenONE times out with a timeout prompt (“Maximum number of conditioning pulses reached”) but you think the current cell suspension is still ok, click **Continue**.
        2. Watch for situations requiring intervention, including: (details below)

| **Issue(s)** | **Action** | **Remedy** |
| --- | --- | --- |
| Morphology thresholds need adjustment | Let run continue | Adjust thresholds, takes effect immediately |
| Cells not tracking well, or focus is poor | Pause run | Adjust focus and/or repeat background generation and mapping, resume run |
| Residual liquid on nozzle tip | Pause run | Water dip and/or adjust nozzle parameters, set nozzle parameters, resume run |
| Cells too concentrated, clumpy or dilute, need to change cell suspension | Abort at next timeout | Reset settings, adjust field, take more cells, repeat CellenONE setup, restart run |

- - 1. To make adjustments outside the CellenONE window mid-run, you will need to pause the run when the system has *not* found a droplet to spot, or the wrong droplet will be spotted when you resume. To **pause**, do the following:
       1. If CellenONE is still successfully identifiying spottable droplets, set the morphology thresholds so that Max Area is *smaller* than Min Area, which will prevent a spottable droplet from being identified.
       2. Before CellenONE times out, click the yellow **Nozzle Setup** button to queue up a pause once the current step (spottable droplet search) is complete.
       3. If CellenONE does identify a cell instead of timing out, click **Continue** at the Continue/Cancel prompt, then click **Nozzle Setup** again. Do not proceed with adjustments until you reach a timeout.
       4. When you get a timeout prompt, click **Continue**. The run will now be in a safely paused state, and you can make adjustments to the Nozzle Setup tab of the main Scienion AG software.
       5. To adjust focus while the run is paused:
          1. Set LED Pulse [µs] to **3**, **Autofocus**, then set LED Pulse [µs] back to **7 OR** manually adjust focus using the Back/Front step controls.
          2. If you wish to continue with the same background image and mapping, click **Test Droplet** in the CellenONE window and check if cells are being correctly tracked. If not, repeat focus adjustment.
          3. If you wish to get a new background, turn Print Run Data File **Off**, repeat background generation, repeat mapping, then turn Print Run Data File back **On**.
          4. If you manually adjusted the focus, click **Set Nozzle Parameters**.
       6. To adjust mapping, first turn Print Run Data File **Off**, repeat mapping, then turn Print Run Data File back **On**.
       7. When done with adjustments, click **Continue** at the Continue/Cancel prompt to resume the current run.
       8. Reset CellenONE morphology thresholds before the next timeout.
    2. If the cells in the nozzle are either too concentrated, clumpy or dilute, you will need to abort the run, refresh the cells and start another run:

***DO NOT pause and use Nozzle Setup operations to pick up more cells mid-run, as the safe position override will cause the nozzle to crash into the strobe LED. Instead:***

- - - - 1. At timeout, determine the last row/column position spotted, either from the log file and “Printed” images in the run data folder where the images and cell data are recorded, or from the X Pos. and Y Pos. values in the Run tab. *Note: the X Pos. and Y Pos. values start at 0, so add 1 to get the actual row and column numbers, then go back one well in the spot order since the position shown will be the one about to be spotted.*
        2. Click **Abort** to stop the run, turn Print Run Data File **Off,** and close the CellenONE window.
        3. **IMPORTANT:** Reset the SafePosition Z value to **19000** *before moving the nozzle to the reagent plate* (see step 4.5). Double check that the value has been set correctly for the correct position.
        4. Adjust the field setup to clear the wells that got spotted.
        5. Add more cell suspension to the reagent plate, then repeat cell aspiration and drop check.
        6. With the nozzle at the camera position, set the SafePosition Z value to match the Target Z value. Ensure that the warning sticky note is in place over the **Nozzle Setup** tab.
        7. Do task **Cell_Monitor** again, and repeat background generation and mapping, then turn Print Run Data File back **On**.
        8. Start run **CellenOne_Chiller** again with the same Print Run Data File ID. Images and spotted cell data will continue to be added to the same folder and log file.

Summary of run settings for CellenONE setup and well spotting stages:

| **CellenONE stage** | **Directory** | **Save Images** | **Print Run Data File** | | **Negative Control** | **Field file** | **# drops** |
| --- | --- | --- | --- | --- | --- | --- | --- |
|  |  |  | **Box** | **Text** |  |  |  |
| Test Droplet | Run folder | Off | Off | Chip/library ID | Off | N/A | N/A |
| Get Background | Run folder | **ON** | Off | Chip/library ID | Off | N/A | N/A |
| Mapping | Run folder | Off | Off | Chip/library ID | Off | N/A | N/A |
| Cell wells | Run folder | Off | **ON** | Chip/library ID | Off | Custom | **1** |
| No-cell control wells | Run folder | Off | **ON** | Chip/library ID | **ON** | Custom | **1** |
| Corner wells | **delete_me** | Off | Off | Chip/library ID | Off | SmartChip-empty- 1field-3corners | **10** |

- 1. If desired, start run **CellenOne_Chiller** again to spot no-cell control (NCC) wells, according to the above summary table. The negative control option used here causes droplets *containing no cells* to be spotted.
  2. If performing chip imaging of fluorescent labeled cells, start run **CellenOne_Chiller** again to spot corner wells to be used for corner identification on the microscope, according to the above summary table.
  3. Settings reset and CellenONE cleanup
     1. Once all CellenONE spotting is complete, turn Print Run Data File **Off**, set Negative Control to **Off** and set Directory for Run Folders to ***Desktop\delete_me***.
     2. Close the CellenONE window

- - 1. **IMPORTANT:** Reset the SafePosition Z value to **19000**. Double check that the value has been set correctly for this position, then take down the reminder note.
    2. Set the LED Pulse [µs] back to **3**.
    3. Remove blackout panels from the spotter enclosure.
    4. Check the nozzle is at the camera station, then go to the **Pump** sub-tab and set **Flush**, **25 µL**, and **10 µL/s**, then click **Start** to pump any remaining cells and PBS out into your cell collection tube. If you want to keep these cells, home the spotter, retrieve the collection tube and replace it with another tube for waste collection.
    5. Clean and dry the nozzle, perform run **Wash_NozzleRemoval_SciCleanTray1**, selecting any target position(s).
    6. Exit the CellenONE version of the Scienion AG software. Use the regular (sciFLEX_S3) version for all other spotting operations.

1. **RNA fragmentation**
   1. Seal chip with Bio-Rad MicroSeal A film.
   2. Quick-spin chip for 30sec in clean room VWR centrifuge.
   3. Place chip in thermal cycler and run program **RNAfrag** to fragment RNA:

| Lid 100 °C | |
| --- | --- |
| Target temp | Time |
| 85 °C | 6 min |
| 4 °C | Hold |

- 1. Hard-spin chip for 2 minutes at 4000 rpm in benchtop centrifuge.
  2. Place chip on chiller adapter, wait ~15 seconds, then remove sealing film.
  3. During the incubation period, set up the spotter as follows:
     1. Prepare fresh system liquid, fresh water and sciCLEAN 8, and empty waste carboy and flush bottle. If necessary, refill and remount the humidifier.
     2. Re-prime PDC 70 Type 4 nozzle, and ensure you have a stable drop with water before proceeding.

1. **Reverse transcription**

The reaction set up per well is:

|  | **Reagent (nano L)** |
| --- | --- |
| SMART scTSO Mix | 4.5 |
| RNase Inhibitor | 0.5 |
| SMART scN6 | 1 |
| DEPC H20 | 7.7 |
| 1% TWEEN | 0.8 |
| SMARTScribe RT | 2 |
| TOTAL | 16.5 |

- 1. Hard-spin the chip for 2 minutes at 4000 rpm in benchtop centrifuge.
  2. Place chip on chiller adapter, wait ~15 seconds, then remove sealing film.
  3. Spot the reverse transcription (RT) mix:
     1. Load field file for the RT mix spotting.
     2. Load RT mix into reagent plate (aspirate volume + at least 3 µL).
     3. Aspirate RT mix using **TakeProbe** task (spot volume + at least 5 µL).
     4. Optimize voltage & pulse, **Set Nozzle Parameters**, and **Autofocus**. Record voltage and pulse values.
     5. If necessary, perform the **Nozzle Head Cam Wizard**.
     6. Get drop volume and enter into calculator to find number of drops to spot.
     7. Use “Edit Field Table…” dialog to set number of drops in the field file.
     8. Start a **SpotRun_Chiller** run on the appropriate target position.
     9. Within 1-2 rows of spotting after FTRP chip registration, and periodically throughout the run, check drop stability and volume.
     10. If chip wells were split for multiple aspirates, repeat the above steps to spot additional wells with different field files.
     11. When spotting is complete, execute a **WFS_sciCLEANtray1_WFS** task to clean the nozzle. Note: if nozzle parameters were changed significantly for reagent dispensing, sciCLEAN 8 and/or water may not spot optimally.
     12. Perform run **Wash_NozzleRemoval_SciCleanTray1**.
  4. RT incubation:
     1. Seal chip with Bio-Rad MicroSeal A film.
     2. Hard-spin chip for 2 minutes at 4000 rpm in benchtop centrifuge.
     3. Place chip in thermal cycler and run program **RT**:

| Lid 100 °C | |
| --- | --- |
| Target temp | Time |
| 42 °C | 60 min |
| 70 °C | 10 min |
| 4 °C | Hold overnight |

- - 1. Hard-spin chip for 2 minutes at 4000 rpm in benchtop centrifuge.
    2. Spotter shutdown (overnight):
       1. Perform run **Wash_NozzleRemoval_SciCleanTray1**.
       2. Do task **7_DayPurge** to dry the system.
       3. Empty sciCLEAN 8 from the wash tray, then rinse the tray with particle-free DI water.
       4. If the humidifier was used, empty it and leave it to dry.
       5. The software and power can be left on so that the chiller stays at temperature.

**DAY 2:**

1. **Index Primer Spotting**

PCR primers the RT reaction wells. The required volumes of wash solutions will depend on the number of primers being spotted, with large numbers of primers often requiring topping up of the wash trays. Four nanolitres each of the 6.25 uM stock I5 and I7 index primers are spotted per well.

- 1. Set up spotter with the following details/modifications:

| **Setting** | **Spotter setup for Primer Spotting** |  | **Also check:** |
| --- | --- | --- | --- |
| Software version: | Standard |  | Humidifier filled & connected if needed |
| Directory: | Dedicated run folder |  | SafePosition Z=19000 |
| Target type: | SmartChip_Chiller |  | Probe = Chilled_Armadillo_384_well_plate |
| FTRP check run: | FTRPcheck_Chiller |  | System liquid attached with sipper down |
| Spotting run: | Primers_SciCleanTray2L_TweenTray2R |  | Waste carboy emptied |
| Chiller temperature: | Dew point +1°C if pre-spotting, or dew point –1°C if adding to active wells |  | Flush bottle emptied |
| Fresh water level: | At least 2/3 full |  | Collection tube below camera |
| sciCLEAN 8 wash: | 5mL 1% sciCLEAN 8 in tray 2L |  | Nozzle primed, drop checked & focused |
| Tween wash: | 5mL 2% Tween 20 in tray 2R, plus topup |  | Nozzle head camera wizard run |
| Nozzle: | PDC70 Type 1 |  |  |

- 1. Primer plate setup:
     1. Aliquot 8 to 15 µL of primers to a clean Armadillo 384-well plate.
     2. Aliquot 8 to 15 µL of one primer (or pooled mix) to an empty well for drop testing. If setting up the plate for multiple spotting days, include one test well per day.
     3. Seal the plate then spin down to remove bubbles.
     4. Unseal wells to be spotted immediately, and foil-seal wells to save for spotting later.
     5. Place the primer plate on the spotter’s reagent plate chiller adapter.
  2. Chip setup:
     1. In the clean room, extract the SmartChips from their packages. Open the packages carefully and keep them for storing the spotted chips.
     2. Record the chip number of all chips in the batch. This is printed on both the top and bottom of the chip.
     3. Remove the clear film from the front of each chip.
     4. Place chips in the 4x5 chip array on the chiller/vacuum array with the shaded corner at back left and the “Wafergen #####” text at the back. Make sure the array adapter is seated firmly to the front left corner of the array and all chips are seated firmly to the front left corner of the adapter.
     5. Start run **FTRPcheck_Chiller**, selecting the desired chiller target position(s). The spotter will attempt to identify the back left and back right corners of each chip.
     6. If a chip corner image appears with a single red box centered on the top left corner *for all chips*, proceed to step 7.4.
     7. If instead you receive a prompt reporting "Following target(s) will not be produced", click **Abort** then **OK**, check that the array adapter and all chips are seated tightly and squarely to their front left corners, then restart the run.
     8. If chip registration still fails, use the FTRP window to regenerate the corner registration images for the SmartChip_Chiller target type:

| Ref. # | Corner | X Offset | Y Offset | Image filename |
| --- | --- | --- | --- | --- |
| 1 | Back left | 0 | 0 | *smartchip_chiller_GSC.bmp* |
| 2 | Back right | 38482 | 0 | *smartchip_chiller_GSC-2.bmp* |

- - 1. Repeat the FTRP check run to confirm that the chips are all recognized.
  1. Drop optimization (**Nozzle Setup** tab):
     1. Set expected good Voltage [V] and Pulse [µs] parameters for dispensing primers with your mounted nozzle.
     2. Aspirate test primer using a **TakeProbe_05ul** task and check for a stable drop (see steps 3.3 and 3.4 for details). Autofocus.
     3. Get drop volume. Adjust voltage and pulse values to try to get the drop volume as close as possible to a volume that divides evenly into your total desired volume.
     4. Click **Set Nozzle Parameters** and confirm that the correct values are saved in the Nozzle Parameters table.
     5. Perform the **Nozzle Head Camera Wizard** to optimize drop positioning.
     6. Do task **WFS_sciCLEANtray2L_WFS** to clean out the nozzle.
  2. Load and adjust the field file (**Target Setup** tab):
     1. Load the field file for your primer setup.
     2. If necessary, adjust the number of drops in the field file to give the desired total volume, according to # drops = desired volume / drop volume.
  3. Start the primer spot run:
     1. Turn on the chiller vacuum pump to hold the chips firmly in place.
     2. Start run **Primers_GSC_SciCleanTray2L_TweenTray2R**, selecting the desired chiller target positions. The spotter will attempt to identify the back left and back right corners of each chip, then will begin the spotting run.
     3. If a chip corner image appears with a single red box centred on the top left corner *for all chips*, proceed to step 7.6.5.
     4. If instead you receive a prompt reporting "Following target(s) will not be produced", click **Abort** then **OK**, check that the array adapter and all chips are seated tightly and squarely to their front left corners, then restart the run.

- - 1. Once chip recognition succeeds, you will be prompted for the reagent plate, then spotting will begin.
  1. Monitor the run:
     1. Watch the first few spotting cycles to check that spotting proceeds smoothly.
        1. Watch the drop when it *first* spots at the camera after aspirating a primer. If it looks poor, *immediately* click **Nozzle Setup** to pause before auto drop detection, as autodrop failure will cause the aspirated primer to not be spotted. Adjust the drop, click **Set Nozzle Parameters**, then **Continue**.
     2. Periodically throughout the run, check the following:
        1. Dewpoint: Chiller should stay at 1°C above dewpoint, or as set.
        2. Drop volume & stability (Autodetected Drop Positions graphs): If values deviate significantly, adjust nozzle parameters as needed and/or repeat autofocus. Drop detection is done both before and after each primer is spotted, and gaps or values of 0 in these plots indicate failure to detect a droplet at one of these steps.


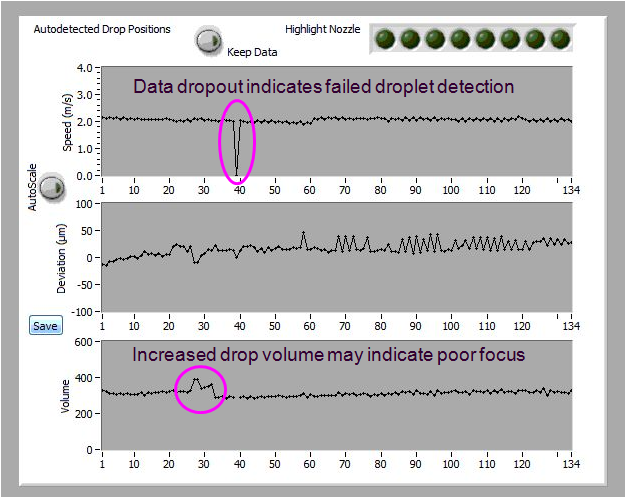


Figure 9: Autodetected Drop Positions graph

- - - 1. sciCLEAN & Tween liquid levels: Top up during water wash steps
      2. System liquid level: Prop up bottle if below sticker on pump tower
      3. Fresh & waste bottles: Pause run and refill or empty if needed
  1. When the run is complete, click **Save** in the Autodetected Drop Positions graph area to save an image of the graphs into your run folder.
  2. Fill in non-spotted wells after run completion:
     1. When the run is finished, you may see a Not Spotted Probe(s) window indicating failures to auto-detect a droplet at either the pre- or post-spotting points
     2. Pre-Autodrop list: No droplet was detected prior to spotting, so spotting was skipped for these wells. Click **Save…** to create a field file to re-spot these wells.
     3. Post-Autodrop list: No droplet was detected after spotting, so primers may or may not have been spotted for these wells.
        1. Inspect post-autodrop droplet images in your run folder. Images are named with the well position and a date/time string, with the second image for each well being the post-autodrop image. In some cases, a drop will be visible in the image even if not autodected, so these wells were likely spotted fine.
        2. For wells where the images do not show a good droplet, chips can be imaged under cyan illumination to visualize primers in the wells IF the wells were empty prior to primer spotting. Using the expected spotting pattern, you can examine wells that should have ONLY received the primer in question to determine whether each primer was successfully spotted.
        3. In the absence of evidence of good spotting from either post-autodrop images or microscopy, assume post-autodrop failed primers were not spotted.
        4. *Double-click* rows in the well list to select wells for re-spotting. When done, click **Save…** to create a field file to re-spot these wells.
        5. Repeat primer spotting runs for both pre- and post-autodrop failed primers.
        6. Cleanup after completion of spotting.
        7. After all spotting is complete, turn off the vacuum pump.
        8. Wash and dry it by performing run **Wash_NozzleRemoval_SciCleanTray1**, selecting any target position(s).

1. **Index PCR**
   1. Prepare fresh system liquid, fresh water and sciCLEAN 8, and empty waste carboy and flush bottle. If necessary, refill and remount the humidifier.
   2. Prime PDC 70 Type 4 nozzle, and ensure you have a stable drop with water before proceeding.
   3. The reaction set up per well is:

|  | **Reagent (nano L)** |
| --- | --- |
| SeqAmp CB PCR Buffer (2X) | 25 |
| DEPC H20 | 24 |
| SeqAmp DNA Polymerase | 1 |
| TOTAL | 50 |

- 1. Place chip at ROOM TEMPERATURE, remove sealing film and wait ~15 seconds for complete evaporation. The primer spotting had resulted in evaporation and this step ensures all liquid is uniformly removed from all wells. The water volume in the index PCR mix is accordingly adjusted.
  2. Spot PCR mix:
     1. Load field file for the PCR mix spotting.
     2. Load RT mix into reagent plate (aspirate volume + at least 3 µL).
     3. Aspirate RT mix using **TakeProbe** task (spot volume + at least 5 µL).
     4. Optimize voltage & pulse, **Set Nozzle Parameters**, and **Autofocus**. Record voltage and pulse values.
     5. If necessary, perform the **Nozzle Head Cam Wizard**.
     6. Get drop volume and enter into calculator to find number of drops to spot.
     7. Use “Edit Field Table…” dialog to set number of drops in the field file.
     8. Start a **SpotRun_Chiller** run on the appropriate target position.
     9. Within 1-2 rows of spotting after FTRP chip registration, and periodically throughout the run, check drop stability and volume.
     10. If chip wells were split for multiple aspirates, repeat the above steps to spot additional wells with different field files.
  3. PCR incubation:
     1. Seal chip with Bio-Rad MicroSeal A film.
     2. Hard-spin chip for 2 minutes at 4000 rpm in benchtop centrifuge.
     3. Place chip in thermal cycler and run program **PCR**:

| Lid 100 °C | |
| --- | --- |
| Target temp | Time |
| 94 °C | 1 min |
| 5 Cycles of  (98 °C for 15sec; 55 °C for 15sec; 68°C for 30 sec) |  |
| 68 °C | 2 min |
| 4 °C | Hold overnight |

- - 1. Hard-spin chip for 2 minutes at 4000 rpm in benchtop centrifuge.
  1. **Cleanup**
     1. If fewer than 200 wells will be recovered in each recovery aliquot, spot 30 nL of filtered Ultrapure water per well to increase total recovery volume.
     2. Once all spotting is done, perform a **Wash_NozzleRemoval_SciCleanTray1** run.
     3. Execute a **7_DayPurge** task.

1. **Recovery indexed libraries**
   1. If recovering multiple regions separately from the same chip OR saving an unused portion of the chip for a future experiment, heat-seal the chip prior to recovery:
      1. Place a Wafergen sealing film (clear with *white* backing) on the chip and press firmly into place. Leave the chip on the chiller.
      2. On the thermal cycler, place the orange spacer block on top of the chip adapter, and run the **5_HTSEAL** program. Pause the program while the block is heating.

| **5_HTSEAL:** | Lid 70 °C |
| --- | --- |
| Set temp | Time |
| 55 °C | 1 min |
| 10 °C | Hold |

- - 1. Once the lid is hot, place the chip on top of the orange spacer block, and place the paper gasket on top of the chip.
    2. Close the lid and unpause the program to heat-seal the chip for 1 minute.
    3. When finished, open the lid and check that heat sealing was successful. If the film is not firmly attached, repeat the sealing process.
    4. Look for condensation on the sealing film showing filled wells. Use condensation and/or a pre-marked guide chip as a guide to mark the areas to be eluted.
    5. Place the sealed chip upside-down on a hard flat surface, and trim excess sealing film off all 4 edges of the chip.
    6. For *each* recovery region, do the following steps to recover well contents to a tube:
       1. Before recovery, hard-spin the sealed chip *right-side up* for 2 min at 4000 rpm in benchtop centrifuge.
       2. Cool chip on chiller adapter for ~15 seconds.
       3. Cut and remove a portion of the sealing film to expose the desired wells.
       4. Assemble a Wafergen collection module by pressing a clean 0.5 mL collection tube (Ambion Non-Stick) onto the bottom of the collection funnel.
       5. Place the chip *upside-down* in the collection module and load into the centrifuge.
       6. Hard-spin chip for 2-5 minutes at 4000 rpm in benchtop centrifuge to recover and pool the contents of all exposed wells. The recovered volume should be around 70-80% of the total volume spotted into the exposed wells. If significantly less volume is recovered, repeat centrifugation.
       7. Remove the collection tube from the funnel and store.
       8. The collection funnel can be reused between non-overlapping index primer sets, and additional Ambion Non-Stick 0.5 mL tubes can be used as needed.
       9. Repeat steps 6.1.8.1-6.1.8.8 for each recovery region of interest.
       10. If the chip will be used again, hard-spin *right-side up* again once all wells have been recovered, then store at room temperature for subsequent use.
  1. If collecting all the samples on the chip into one pool, heat-sealing is not required:
     1. Hard-spin chip for 2min at 4000 rpm in benchtop centrifuge.
     2. Place chip on chiller adapter, wait ~15 seconds, then remove sealing film.
     3. Assemble a Wafergen collection module by pressing a clean 0.5 mL collection tube (Ambion Non-Stick) onto the bottom of the collection funnel.
     4. Place the chip *upside-down* in the collection module and load into the centrifuge.
     5. Hard-spin chip for 2-5 minutes at 4000 rpm in benchtop centrifuge to recover and pool the contents of all exposed wells. The recovered volume should be around 70-80% of the total volume spotted into the exposed wells. If significantly less volume is recovered, repeat centrifugation.
     6. Remove the collection tube from the funnel and store.
  2. Perform System shutdown

1. **Bead Cleanup of indexed libraries**
   1. Measure the volume of each recovered PCR amplified sample collected in the 0.5 mL tubes.
   2. Top up pool volume to 50 µL with Qiagen’s elution buffer.
   3. Prepare the following consumables:

| Item | | Storage | Instructions |
| --- | --- | --- | --- |
| Aline beads | 2°C to 8°C | | Let stand at room temperature for 30 minutes. Vortex and invert to mix. |
| 70% EtOH | 2°C to 8°C | | Let stand at room temperature for 30 minutes. |
|  |  | |  |

- 1. Add 40 µL of beads and pipette mix ten times.
  2. Incubate for 8 minutes
  3. Place the bead mixture magnetic stand/plate for 5 minutes.
  4. Without disturbing the beads, remove and discard supernatants.
  5. While tubes are still on the magnet, wash the beads with 150 μL of 70% ethanol.
  6. Wait for 30 seconds, then remove the ethanol. Make sure not to remove any beads.
  7. Repeat 70% ethanol wash and removal after 30 seconds for a total of 2 washes.
  8. Air-dry on the magnet for 5 minutes.
  9. Remove from the magnetic stand and add 10 μL Qiagen elution buffer to the beads. Pipette to resuspend.
  10. Incubate at room temperature for 2 minutes.
  11. Place on the magnetic stand for 2 minutes.
  12. Transfer total volume of eluate to a new tube/well.

1. **rRNA depletion**
   1. The reaction mix set up per well is:

|  | **Reagent (uL)** |
| --- | --- |
| Nuclease-Free Water | 6.8 |
| 10X ZapR Buffer | 2.2 |
| ZapR v2 | 1.5 |
| R-probes | 1.5 |
| TOTAL | 12 |

- 1. Add 12 uL of the reaction mix to 8 uL of the purified indexed library.
  2. Pipette mix 10 times.
  3. Incubate as follows:

| Lid 100 °C | |
| --- | --- |
| Target temp | Time |
| 37 °C | 60 min |
| 70 °C | 10 min |
| 4 °C | Hold |

1. **PCR amplification (2^nd^ PCR)**
   1. The reaction mix set up per well is:

|  | **Reagent (uL)** |
| --- | --- |
| Nuclease-Free Water | 26 |
| SeqAmp CB PCR Buffer (2X) | 50 |
| PCR2 Primers v2 | 2 |
| SeqAmp DNA Polymerase | 2 |
| TOTAL | 80 |

- 1. Add 80 uL of the reaction mix to 20 uL of the rRNA depletion reaction.
  2. Pipette mix 10 times.
  3. Incubate as follows:

| Lid 100 °C | |
| --- | --- |
| Target temp | Time |
| 94 °C | 1 min |
| 14 Cycles of  (98 °C for 15sec; 55 °C for 15sec; 68°C for 30 sec) |  |
| 68 °C | 2 min |
| 4 °C | Hold overnight |

1. **Bead cleanup of PCR reaction**

Follow steps as in 10.4 to 10.15 except that bead amount is 80 uL and elution volume is 20 uL.

1. **Library QC**
   1. Measure concentration of 1 µL library using Qubit dsDNA HS kit on Qubit 4 Fluorometer.
   2. Run 1 µL of library on an Agilent High Sensitivity DNA chip to assess size profile, on Agilent 2100 Bioanalyzer.

**COST ESTIMATE BREAKDOWN**

|  |  | Batch Size | 1000 cells |  |
| --- | --- | --- | --- | --- |
|  |  |  |  |  |
| **Step** |  | ***Reagent Cost per Sample (Can $)** | **hours** | **Hands-on hrs** |
| Cell Spotting (CellenONE) |  | 9.48 | 4.08 | 2.78 |
| WaferGen SmartChip |  | 111.55 | NA | NA |
| cDNA synthesis, indexing, rRNA depletion & PCR |  | 174.06 | 10.00 | 5.5 |
| Bead cleanups |  | 27.73 | 1.50 | 1.50 |
| QC sonicated - spot check on Agilent High Sensitivity Assay (1 chip) |  | 79.92 | 0.50 | 0.50 |
| Qubit QC of libraries |  | 2.20 | 0.25 | 0.25 |
|  |  |  |  |  |
|  | **Total Reagent Cost** | **404.94** |  |  |
|  |  |  |  |  |
|  |  | **Total hours** | **16.3** |  |
|  |  |  |  |  |
|  |  |  | **Total hands-on hours** | **10.5** |
|  |  |  |  |  |
|  |  | *Cost includes shipment and taxes |  |  |
