# Supplementary material for: A Scalable Strand-Specific Protocol Enabling Full-Length Total RNA Sequencing From Single Cells
Source: Front Genet. 2021 Jun 3;12:665888. doi: 10.3389/fgene.2021.665888 (PMC8209500; doi:10.3389/fgene.2021.665888)
Supplement: Supplementary file 5 [file Data_Sheet_1.PDF]

A.

	SMART_v4	Modified RNase H	SMARTer Pico RNA-seq	Exome RNA-seq
**cDNA priming**	Oligo dT	Random	Random	Random
**Fragmentation**	Concurrent fragmentation and adapter addition via transposase	Physiochemical before adding adapters	Physiochemical before adding adapters	Physiochemical before adding adapters
**rRNA depletion**	Oligo dT priming	RNA level RNAse H	Post-cDNA/barcode	Exome capture
**Library construction**	Tagmentation-based	Ligation-based	Integrated into cDNA synthesis	Ligation-based
**Full length**	Mostly	Yes	Yes	Mostly
**One reaction vessel**	No	Yes	Yes	Yes
**Type of RNAs**	Poly(A)	Total	Total	Annotation dependent
**Strand-specific**	No	Yes	Yes	Yes

Input: 0, 0.1, 0.25, 1.0, 5.0, 10.0 ng UHR RNA + spike-ins (in duplicates). Pooled libraries in a HiSeq 2500 lane for assessment.
RNase H: 10.0 ng UHR RNA + spike-ins for reference.

B.

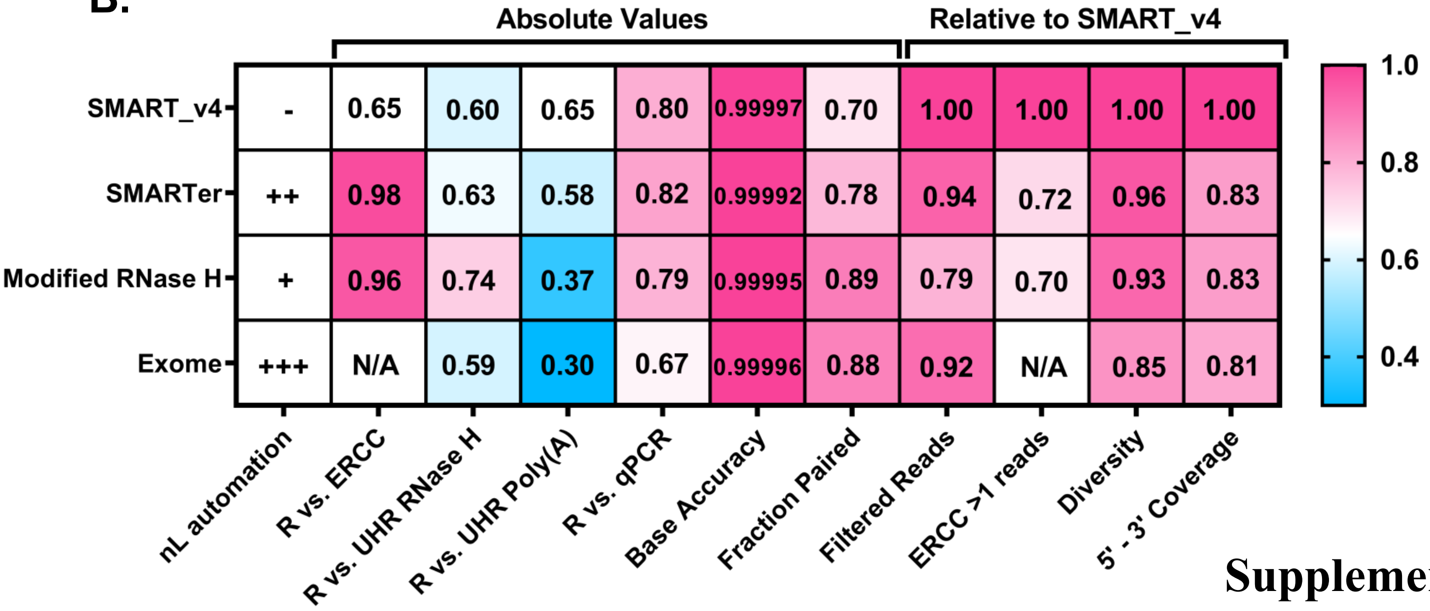

Supplementary Figure 1

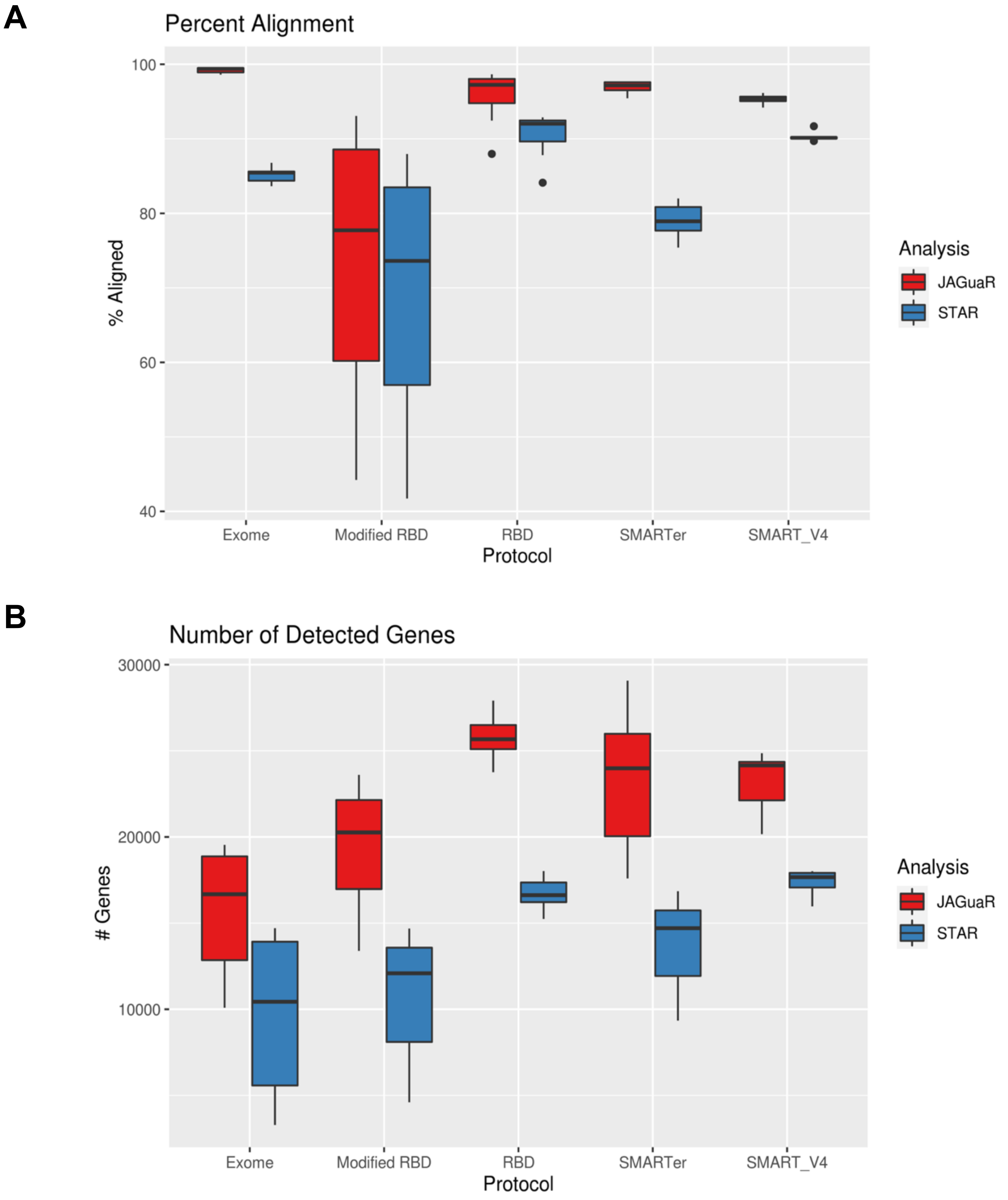

Supplementary Figure 2

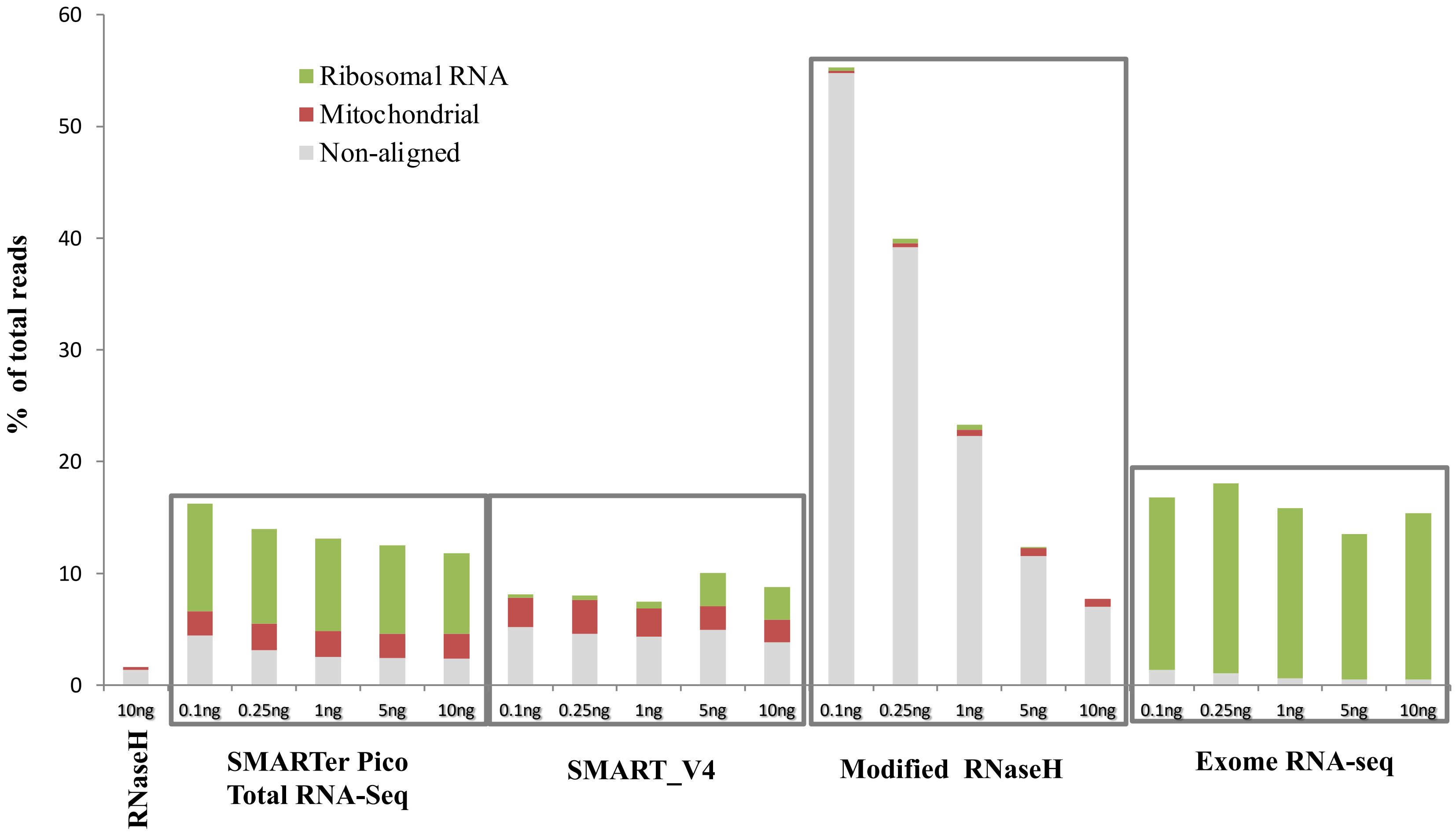

Supplementary Figure 3

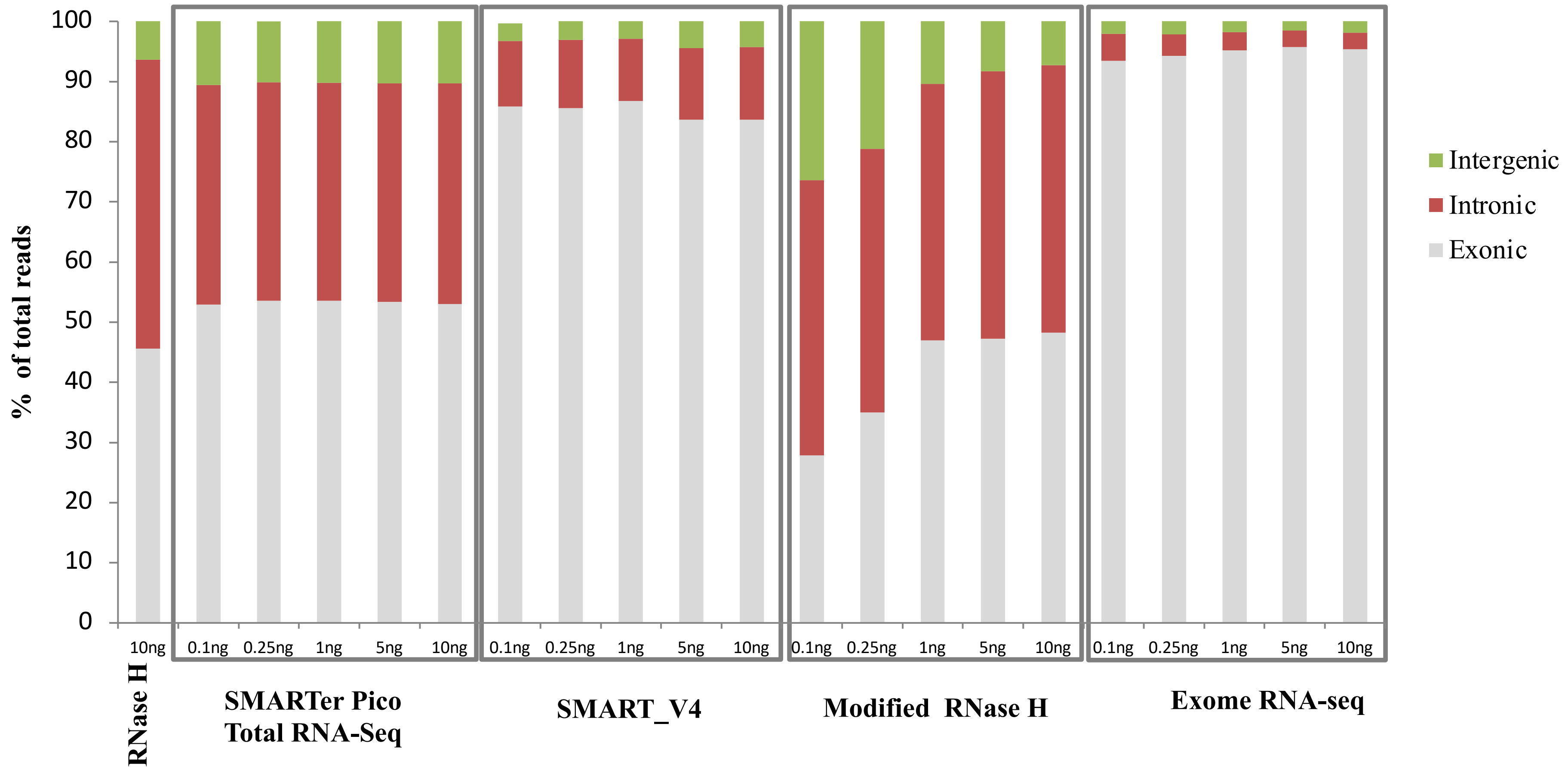

Supplementary Figure 4

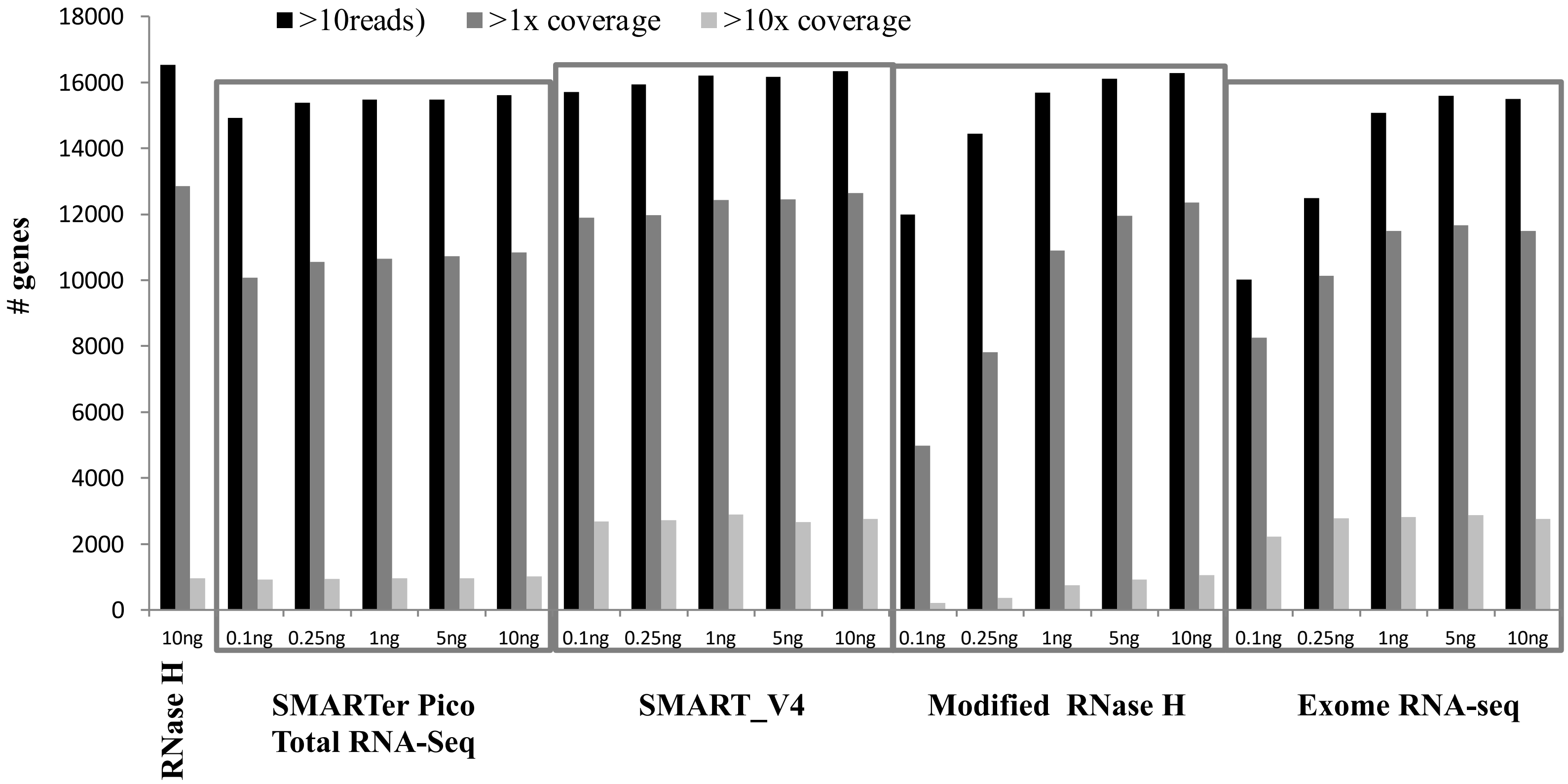

Supplementary Figure 5

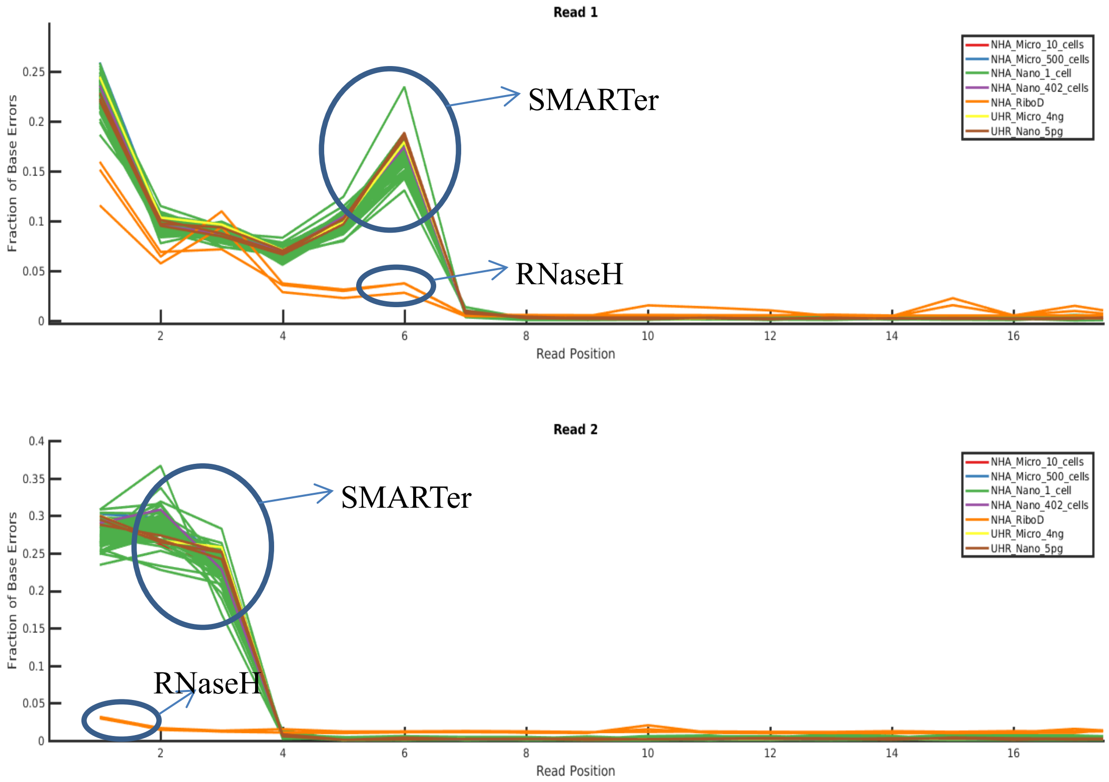

Supplementary Figure 6

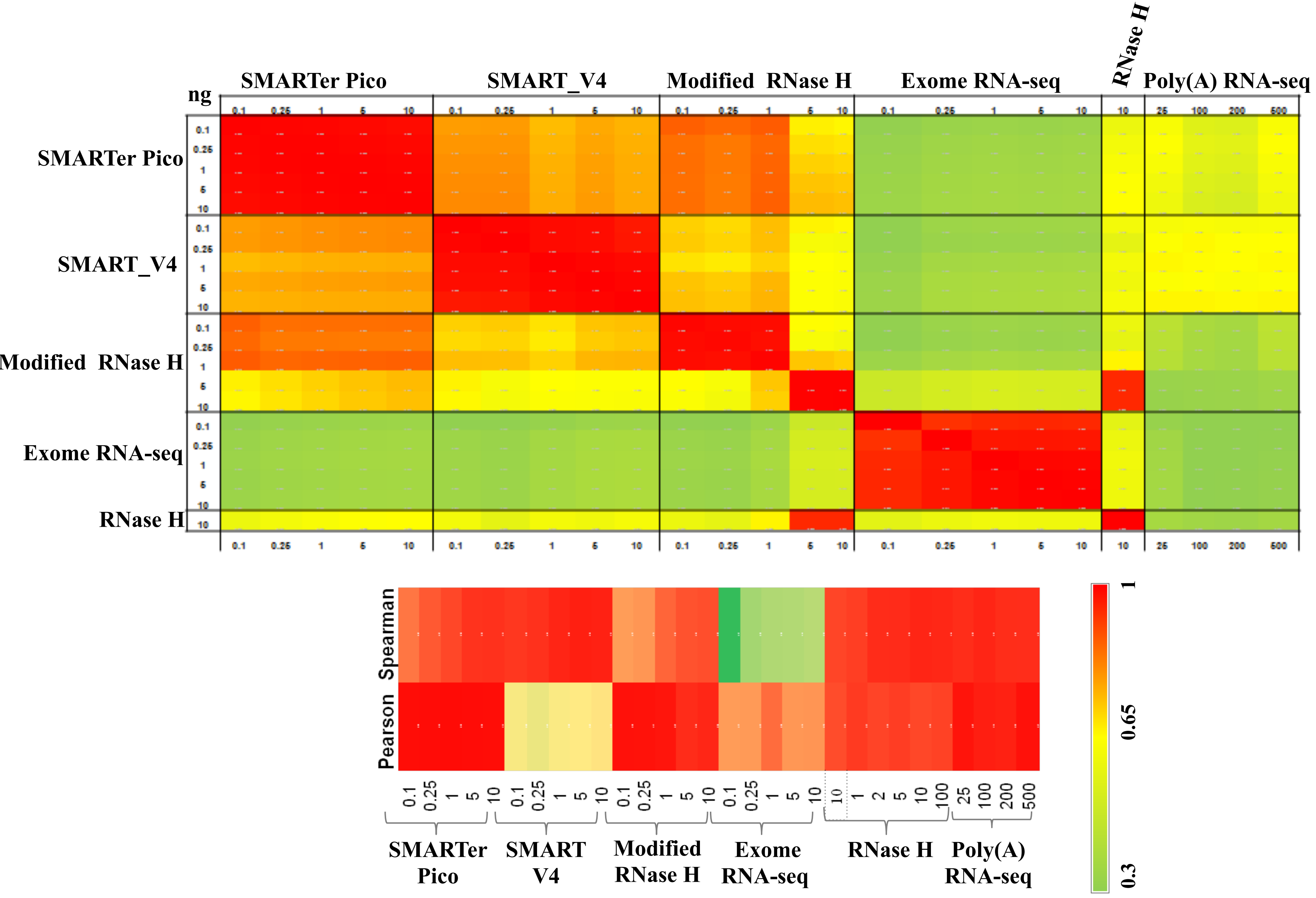

Supplementary Figure 7

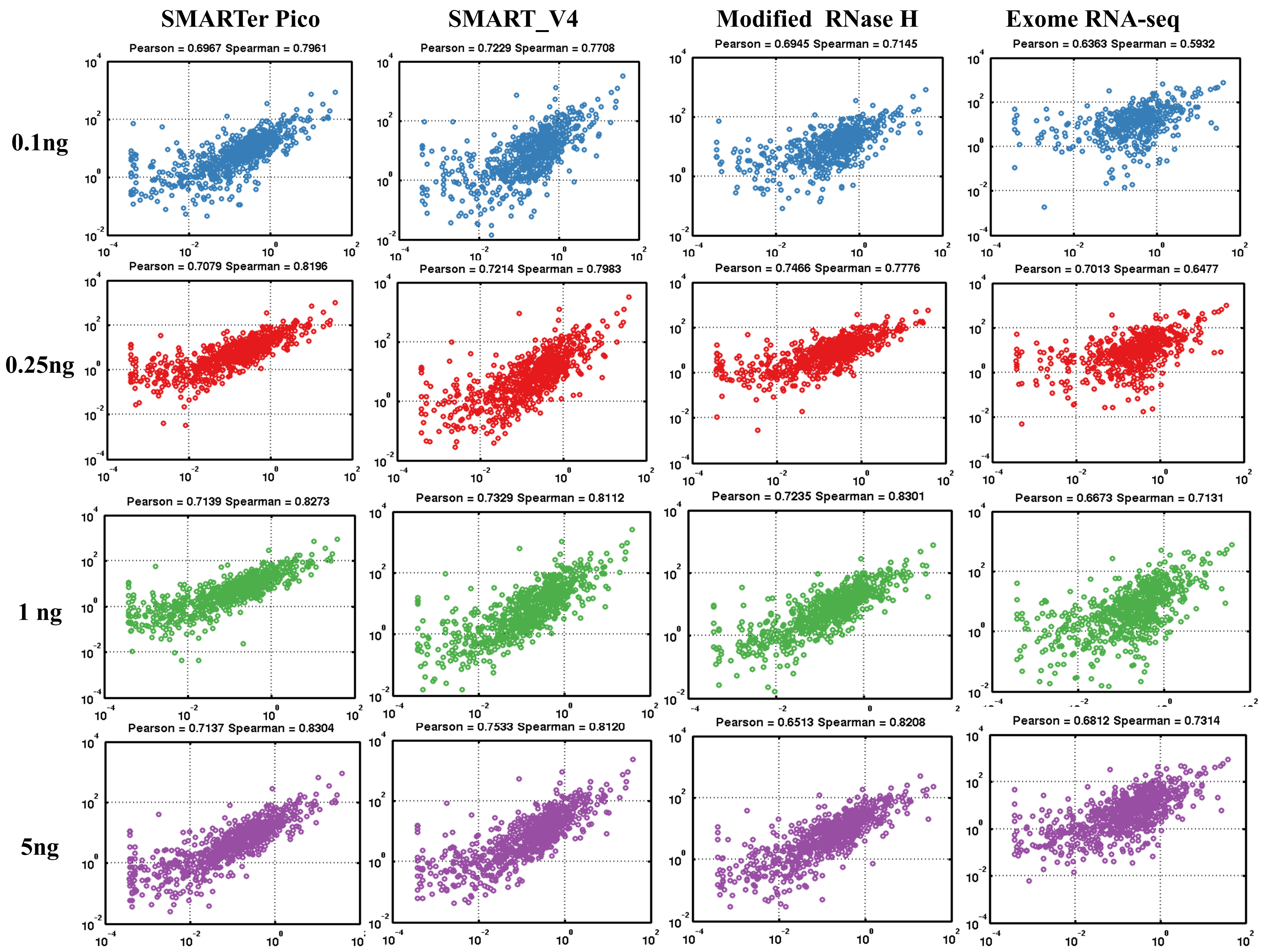

Supplementary Figure 8

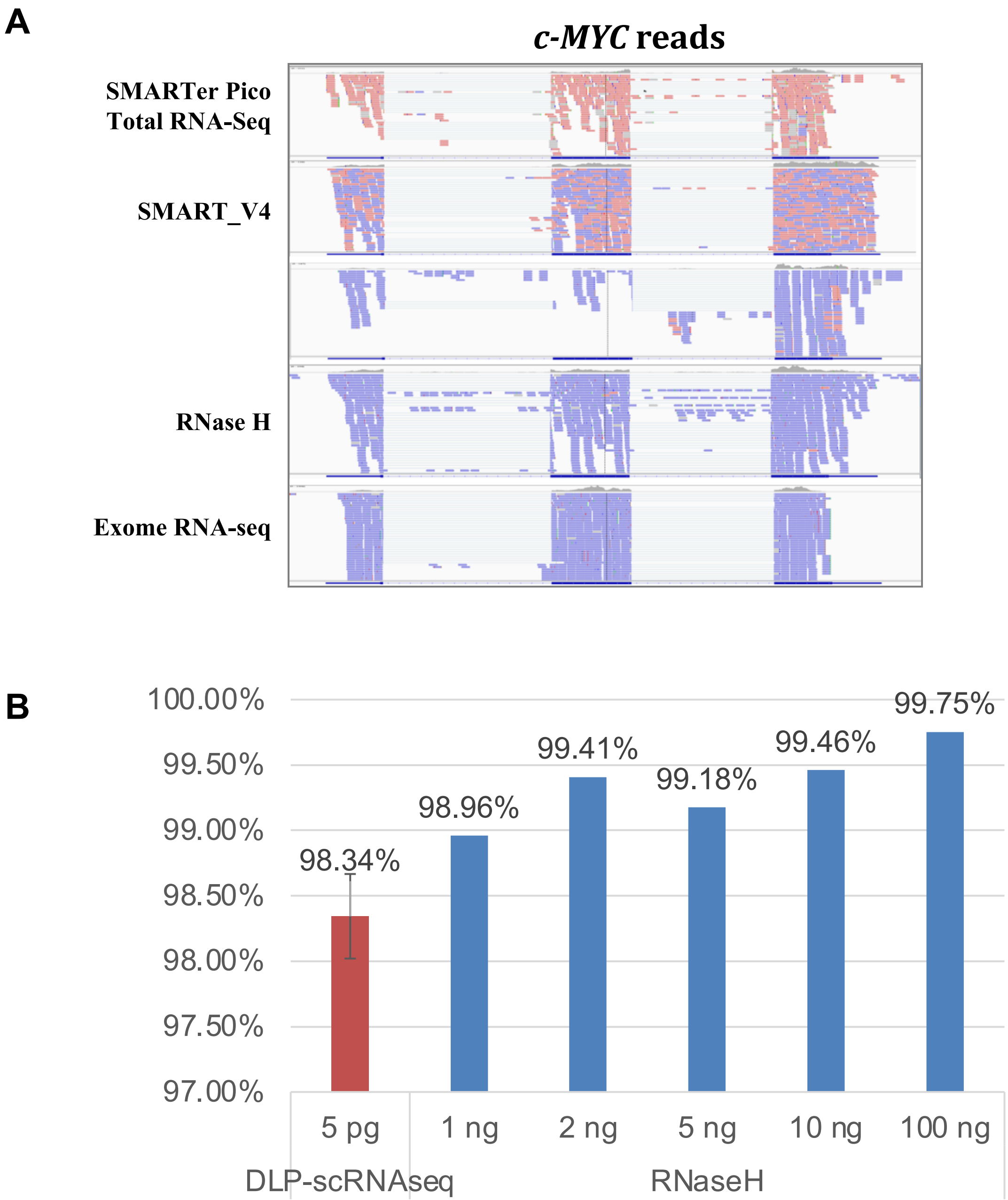

Supplementary Figure 9

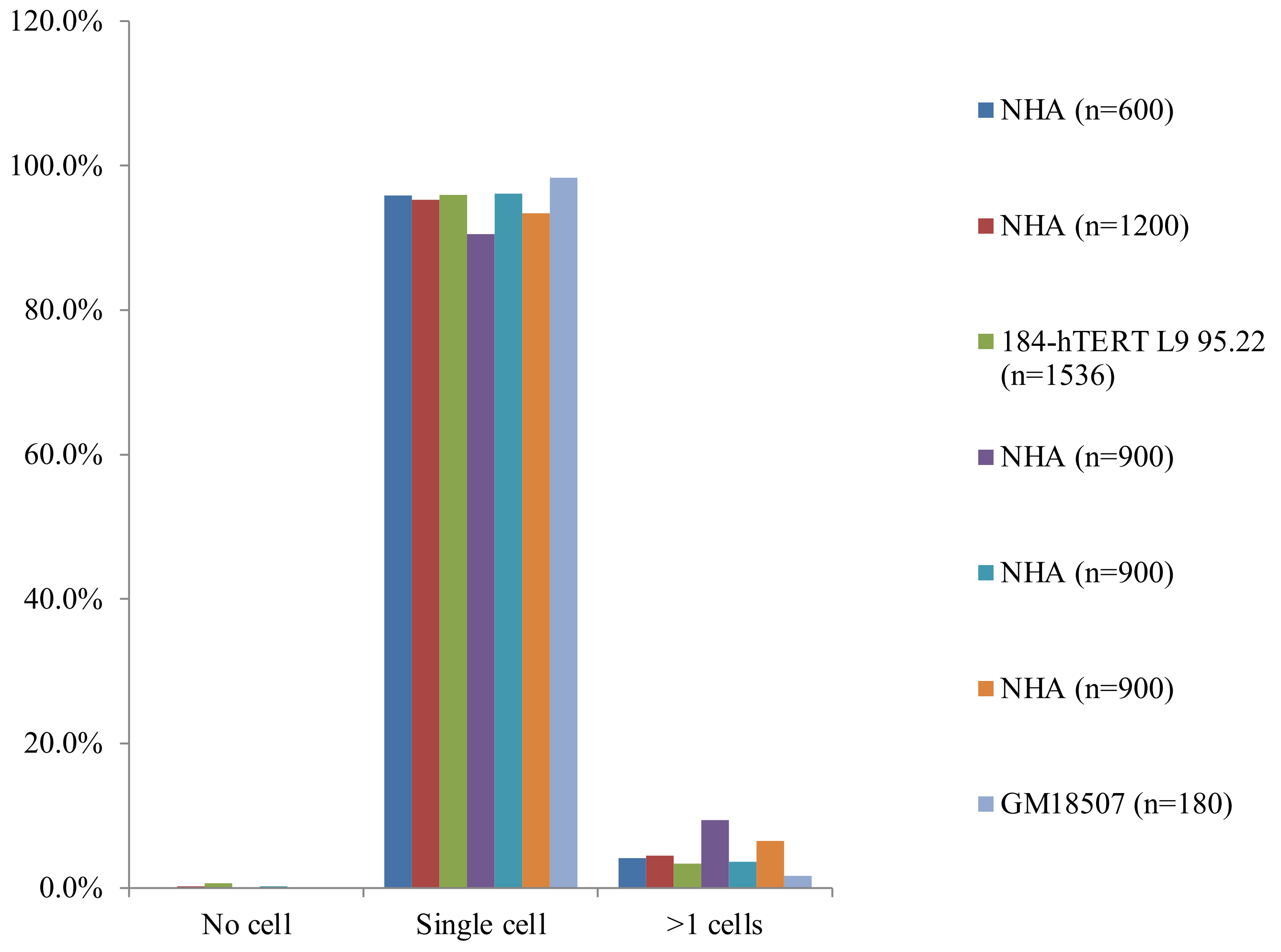

Supplementary Figure 10

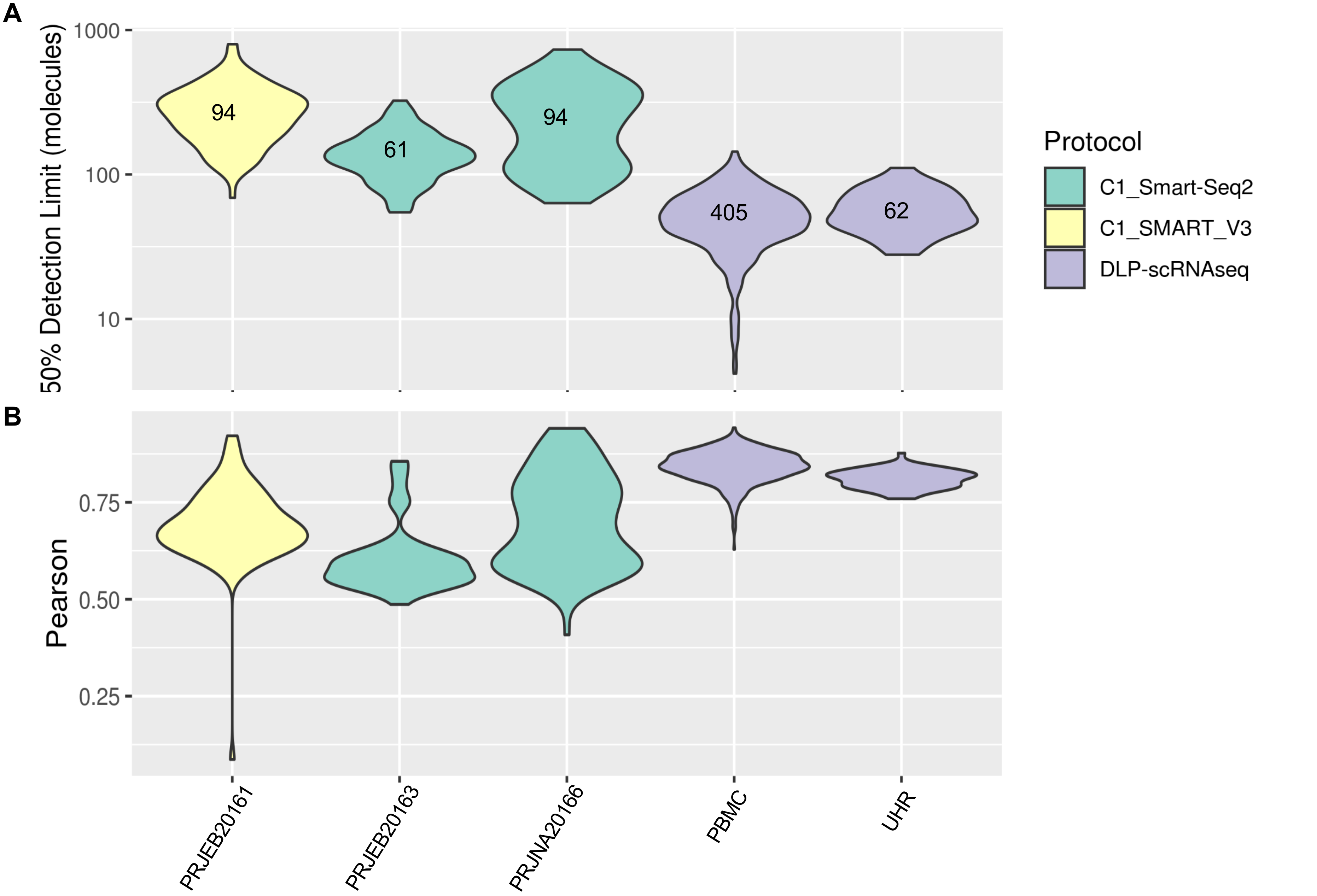

Supplementary Figure 11

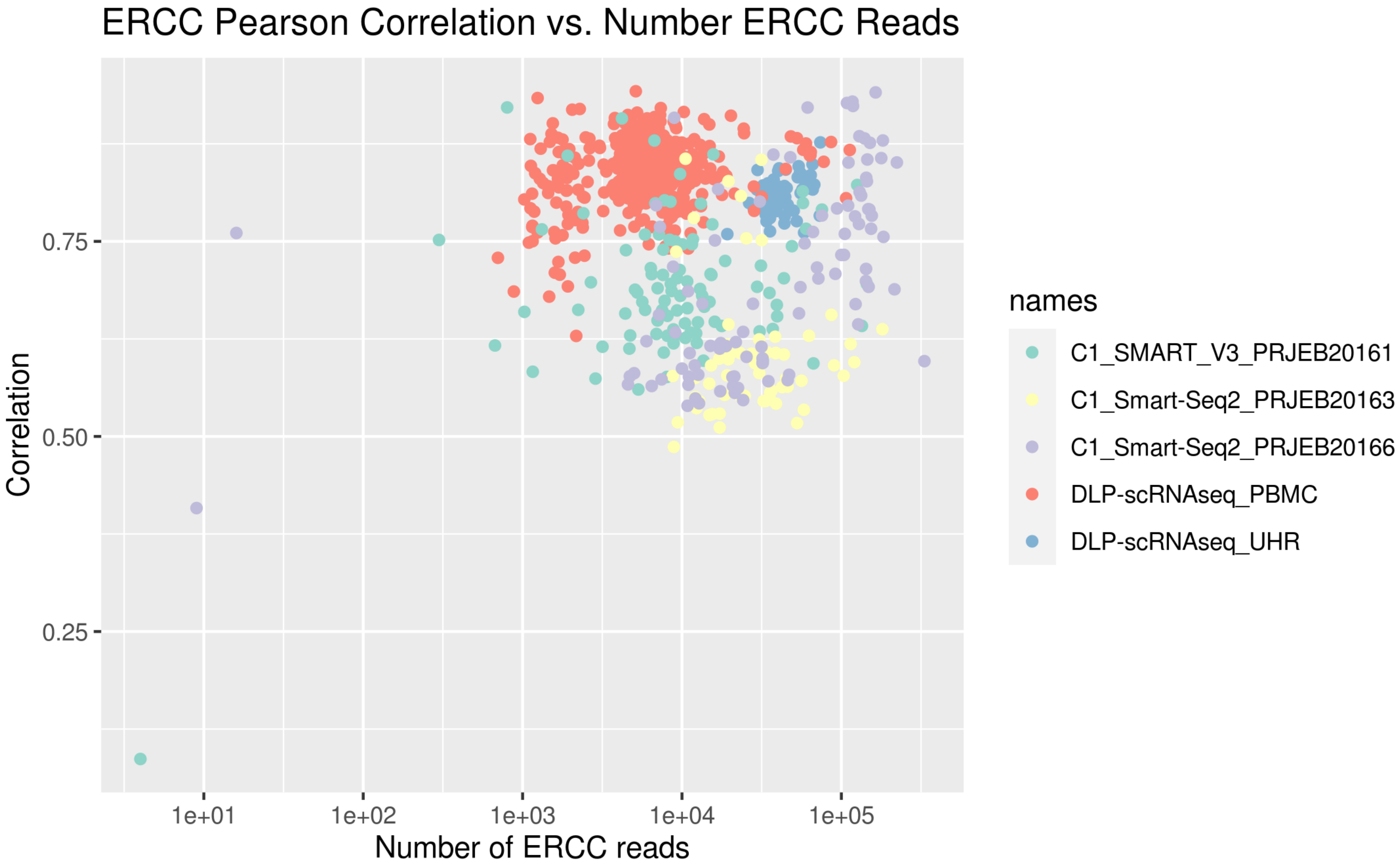

Supplementary Figure 12

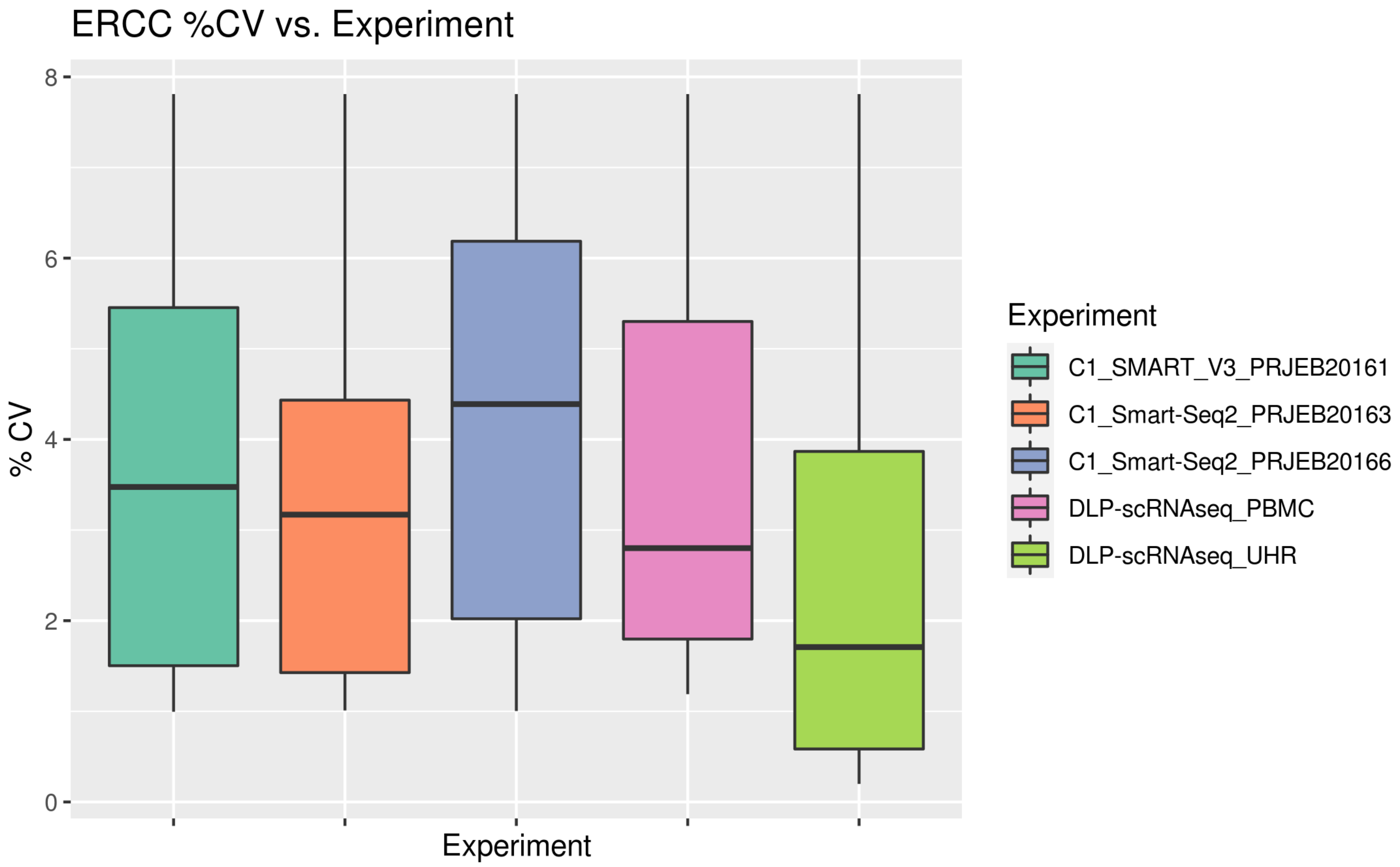

Supplementary Figure 13

A.

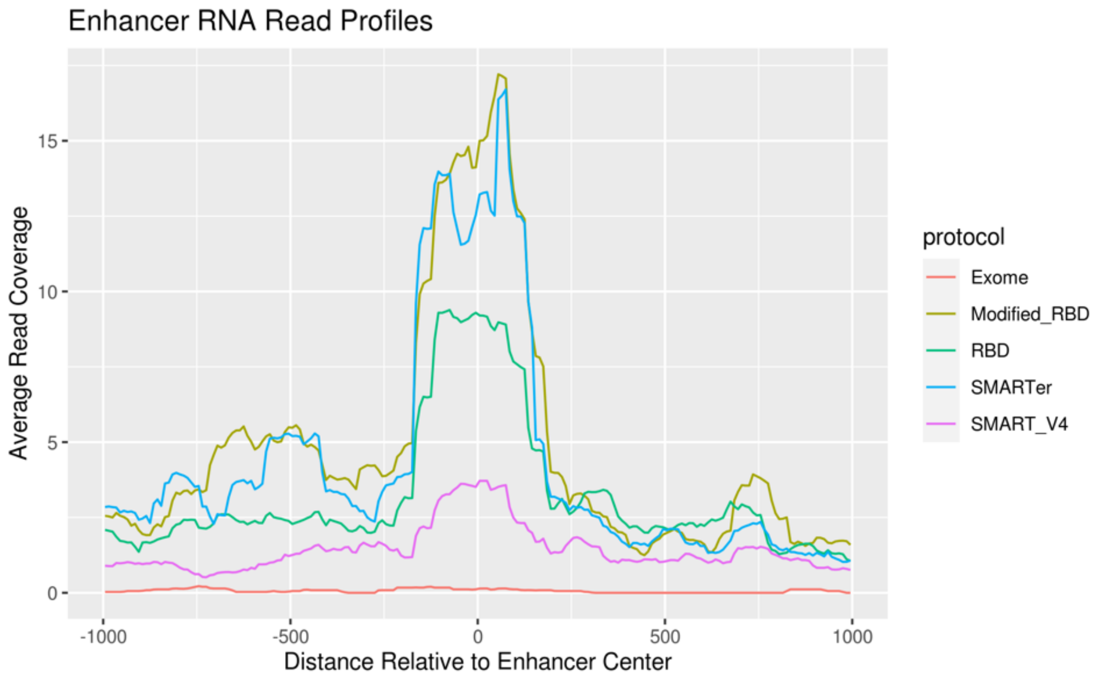

B.

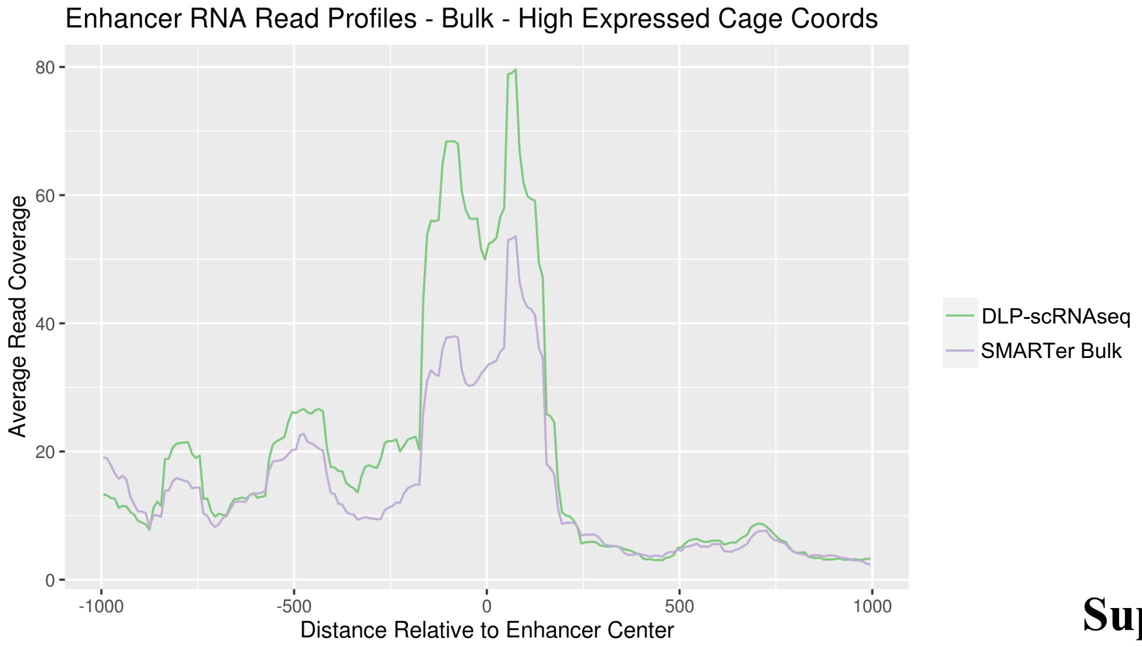

Supplementary Figure 14

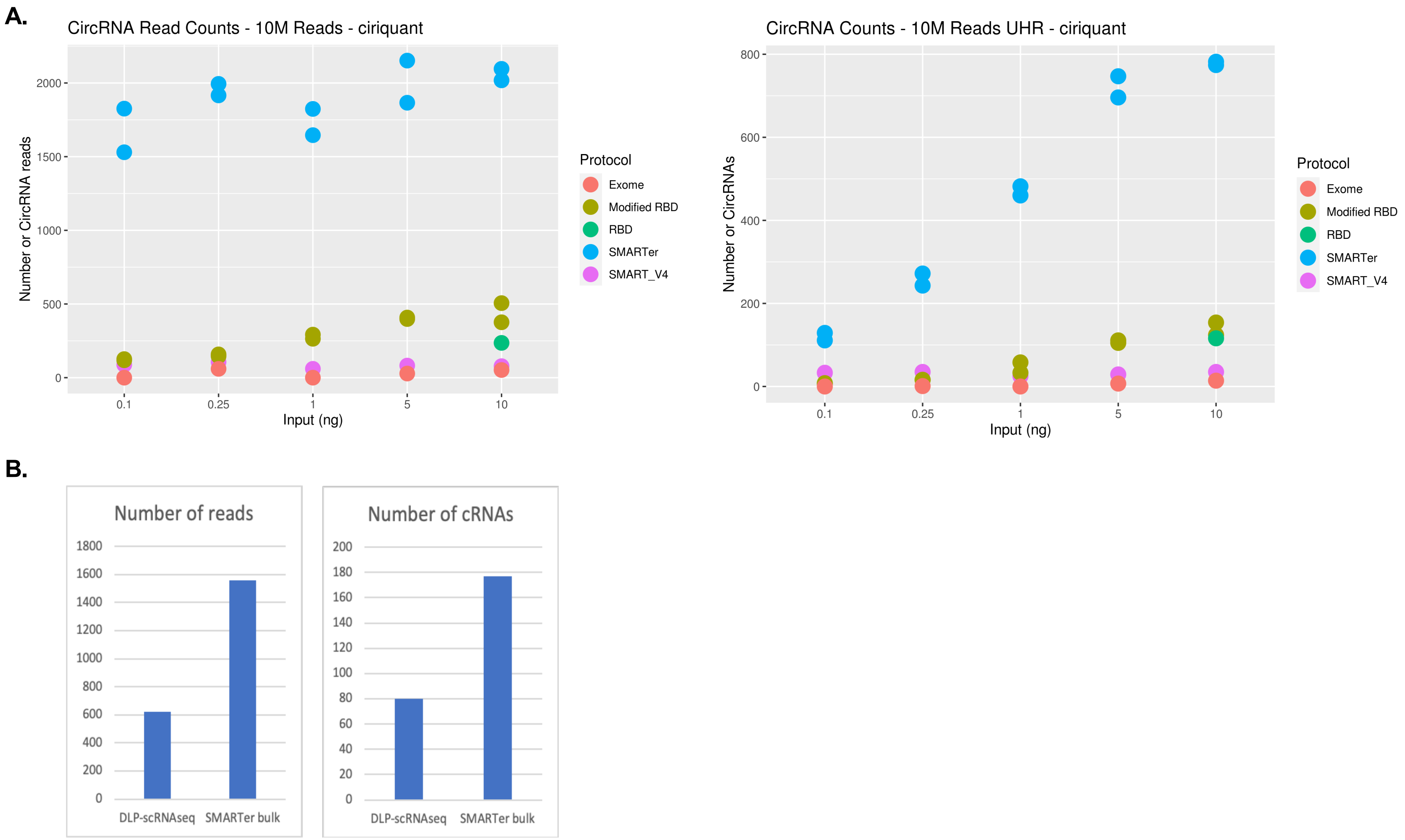

Supplementary Figure 15

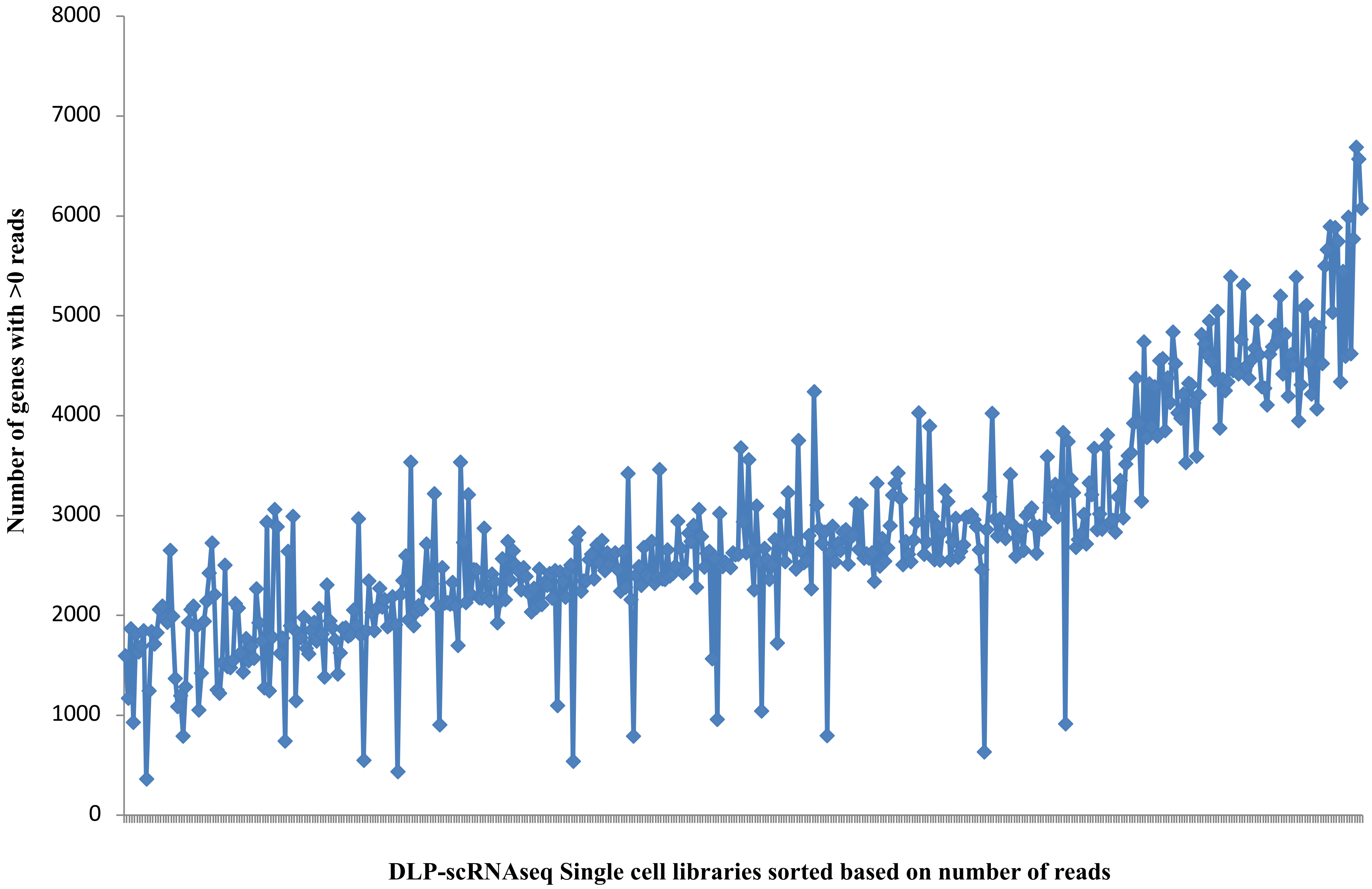

Supplementary Figure 16

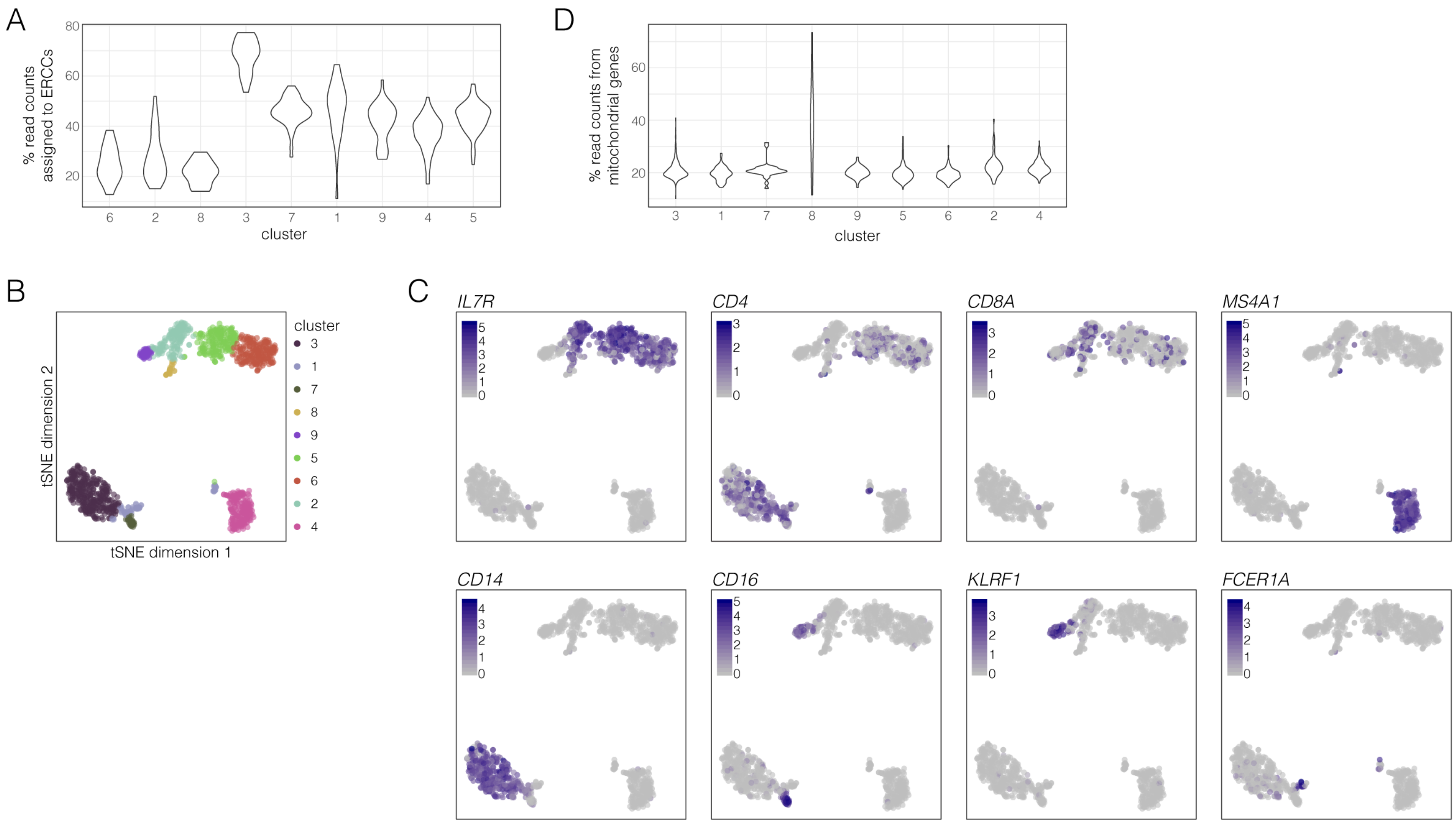

Supplementary Figure 17

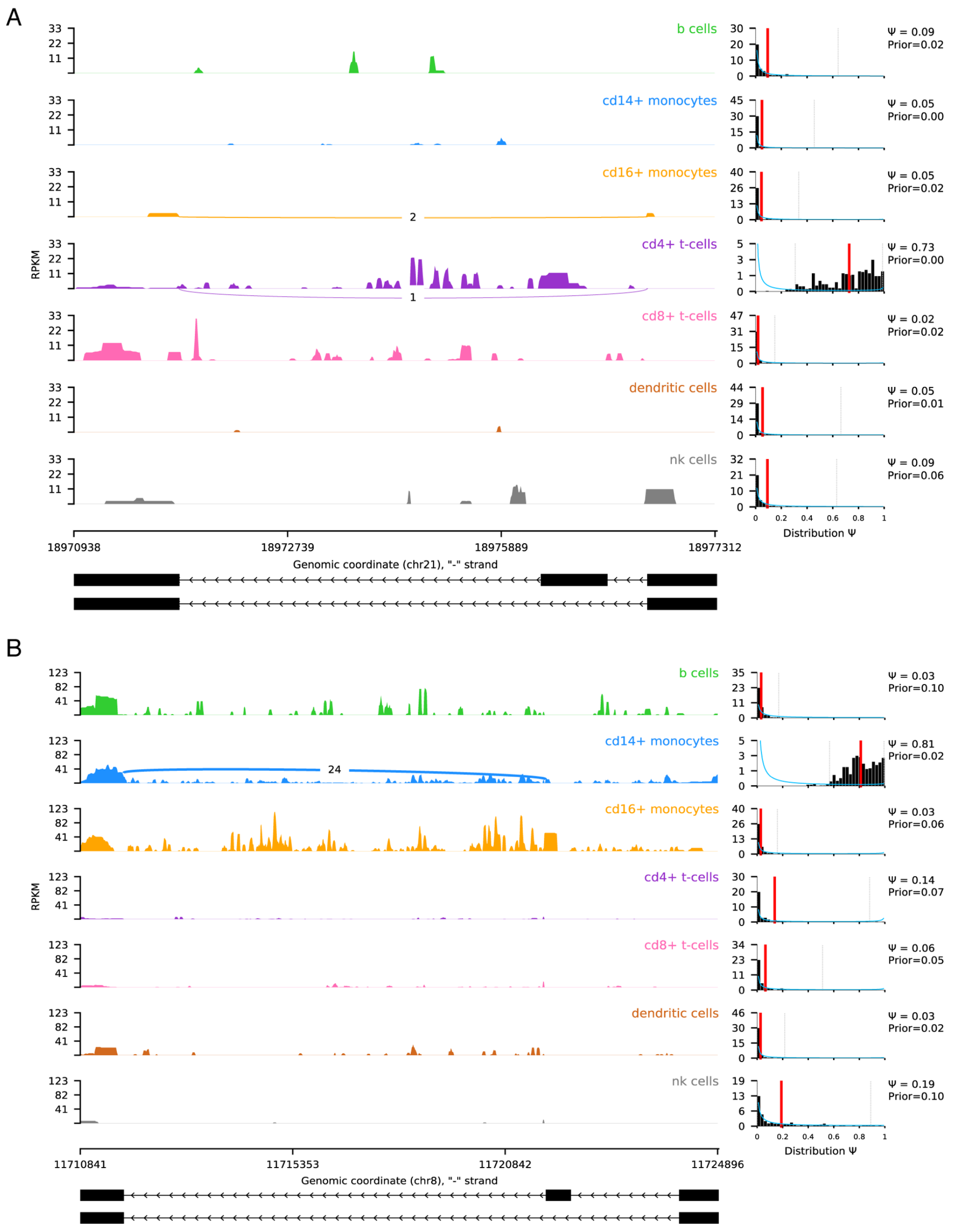

Supplementary Figure 18
